# A novel composite biomarker score for the identification of cognitive impairment in patients with heart failure: a pilot study

**DOI:** 10.1080/1354750X.2025.2585003

**Published:** 2025-12-08

**Authors:** Christina Hoyer-Kimura, Justin Palmer, Radha Gopalan, Kristian Doyle, Jennifer Frye, Elizabeth Juneman, Kristina Irwin, Angelica Galdamez-Avila, Karina Carrillo, Sobeyda Lizzette Cruz, Cindy Schrag, Suzanne Oskouie, Anantharam Kalya, Arianna Bedoya, John P. Konhilas, Nicholas J. Ashton, Lee Ryan, Nancy K. Sweitzer, Meredith Hay

**Affiliations:** a University of Arizona, Tucson, AZ, USA; b Banner Heath Phoenix, Phoenix, AZ, USA; c Banner Heath Tucson, Tucson, AZ, USA; d Sarver Heart Center, Tucson, AZ, USA; e Evelyn F. McKnight Brain Institute, Tucson, AZ, USA; f Banner Sun Health Research Institute, Sun City, AZ, USA; g Banner Alzheimer’s Institute, University of Arizona, Phoenix, AZ, USA; h Department of Psychiatry and Neurochemistry, Institute of Neuroscience & Physiology, the Sahlgrenska Academy at the University of Gothenburg, Molndal, Sweden; i Washington University School of Medicine, St. Louis, MO, USA; j ProNeurogen, Inc, Tucson, AZ, USA

**Keywords:** Heart failure, VCID, cognitive impairment, neurofilament light chain, inflammatory cytokines, biomarkers

## Abstract

**Background:**

Among 6.7 million Americans with heart failure (HF), 40%−60% are estimated to have mild cognitive impairment (MCI) and are at-risk for vascular contributions to cognitive impairment and dementia (VCID). This pilot study examined whether neurodegenerative and inflammatory serum biomarkers are elevated in HF and whether a combination of these biomarkers predicts cognitive performance.

**Methods:**

Thirty-four HF patients (mean age = 69 years, 62% male) were recruited from Banner-University cardiology clinics and underwent blood sampling and neuropsychological testing to derive an ‘Actual Composite Cognitive Score.’ Age-matched healthy controls included (i) 11 individuals (mean age = 63 years, 20% male) who completed identical procedures and (ii) 24 individuals used exclusively for biomarker analysis. Biomarkers—serum neurofilament light chain (NfL), plasma phosphorylated tau (pTau181, pTau217), placental growth factor (PlGF), cytokines, and NT-proBNP—were quantified using Quanterix Simoa, Milliplex, and Elecsys (Roche) assays.

**Result:**

HF participants scored worse in cognitive assessments than controls (*p*=0.0001). Serum NfL (*p*=0.02), IL-6 (*p* < 0.0001), IL-12p40 (*p* < 0.0001), IL-15 (*p*=0.005), MIP-1α (*p*=0.007), TNFβ (*p*=0.03), and TNFα (*p*=0.0002) were increased in HF. NfL and pTau181 correlated with NT-proBNP; NfL, IL-6, and TNFα inversely correlated with cognitive scores. The Composite Biomarker Cognitive Score=NfL+NT-proBNP + IL-6 + TNFα negatively correlated with the Actual Composite Cognitive Score (*r* = −0.60, *p*=0.0002).

**Conclusion:**

These results establish a Composite Biomarker Cognitive Score, which is predictive of cognitive impairment in HF, and may aid in identifying HF patients suitable for cognitive-protective therapies.

## Introduction

There is a close relationship between cardiovascular risk factors and risk for vascular contributions to cognitive impairment and dementia (VCID) and Alzheimer’s disease-related dementias (ADRD). Specifically, in persons with heart failure (HF), there is an increased risk for the development of cognitive impairment and possible progression to VCID and vascular dementia (Vogels et al. 2007; Adelborg et al. 2017; Yang et al. 2021). The mechanisms thought to contribute to cognitive impairment in persons with cardiovascular disease (CVD) include oxidative stress, systemic and brain inflammation (Athilingam et al. 2013b), decreases in cerebral blood flow (CBF) (Alves et al. 2005; Vogels et al. 2007; Sweeney et al. 2018; Montagne et al. 2022) and hypoxia, intracranial atherosclerosis (Pullicino et al. 2008; Lazar et al. cerebrovascular autoregulation (Zuccala 2001), and microembolism (Almeida et al. 2012). In a recent study assessing profiles of cognitive impairment in 140 HF patients, ages 50–85, Miller et al.(Miller et al. 2012) reported that 48% experienced memory impairment. An additional 14% had memory problems plus significant problems in other domains, including executive function and processing speed. HF patients with cognitive impairment show decreased ability to monitor symptoms and carry out self-care activities essential to the management of their disease (Cameron et al. 2012) leading to poorer medication adherence (Alosco et al. 2012), worsening clinical symptoms, more hospitalizations, and higher mortality rates (Dickson et al. 2007; Dodson et al. 2013).

Recently, the American Heart Association published revised diagnostic criteria for VCID (Sachdev et al. 2025). In the clinic, VCID diagnosis and disease monitoring are generally achieved through neurocognitive testing and by magnetic resonance imaging (MRI) scans to detect the presence of cerebrovascular disease, including but not limited to the presence of multiple lacunes, white matter hyperintensities (WMH), and microbleeds that are typically interpreted as a surrogate of cerebral small vessel disease (cSVD) (Prins and Scheltens 2015; Cipollini et al. 2019; Debette et al. 2019; Lennon et al. 2025; Sachdev et al. 2025). However, the assessment and diagnosis of VCID using MRI most often occurs after the development of significant clinically measured cognitive dysfunction, suggesting an urgent need for biomarkers that can identify neuronal and axonal damage in individuals at risk for VCID/ADRD prior to the development of clinical cognitive dysfunction, allowing for early neuroprotective interventions (Hosoki et al. 2023). These needs may be met by identifying a combination of biomarkers specific to HF and cognitive impairment.

Recently, our laboratory has investigated biomarkers for neurodegeneration, including Neurofilament light protein (NfL), and circulating cytokines in our preclinical model of VCID-HF (Hoyer-Kimura et al. 2021) as well as possible treatment therapies with PNA5 (Hoyer-Kimura et al. 2021; 2023). While not specific for VCID, in combination with other biomarkers, NfL may prove effective at identifying at-risk VCID patient populations (Hosoki et al. 2023). In addition, inflammatory processes are known to have an important role in HF pathology and cognitive impairment. In our preclinical studies on VCID, we have shown that HF-related increases in inflammatory cytokines are observed in the brain and circulation (Hay et al. 2019; Hoyer-Kimura et al. 2021). Hence, blood cytokine levels may be potential biomarkers to identify patients at risk of cognitive impairment in HF.

Ideally, if we can predict cognitive impairment in individuals with chronic inflammatory diseases such as HF, we will be able to identify patients at risk for VCID who would benefit from future therapeutic treatment to protect cognitive function. The aim of the present study was to determine if serum biomarkers for neurodegeneration, serum levels of inflammatory cytokines, and biomarkers for cardiac disease (NT-proBNP) are increased in HF patients and if a combination of these biomarkers can predict cognitive performance in HF patients. We hypothesize that a combination of neurodegenerative, inflammatory, and heart disease severity biomarkers together might serve as a composite prognostic biomarker to predict cognitive impairment and risk for VCID in persons with HF and identify those who may benefit from cognitive protective interventions.

## Methods

### Human subjects protection and protocol approvals

The study protocol and patient samples were approved by the University of Arizona Institutional Review Board (IRB). The study was conducted in accordance with the Declaration of Helsinki. All participants gave written informed consent to participate in this study.

### Prospective HF study population and experimental design

Between May 2021 and December 2023, we conducted a prospective study at Banner-University Medical Centers HF clinics (Tucson and Phoenix). We enrolled adults with clinically diagnosed heart failure (HF) (*n* = 34; 61.76% male). All HF participants met the following inclusion and exclusion criteria. Inclusion criteria included clinically diagnosed HF, ≥50 years of age, clinically stable and free from hospitalization for 30 days, and English-speaking. All participants met the American Heart Association (AHA) clinical criteria for HF with or without changes in ejection fraction. Exclusion criteria included active coronary ischemia, significant lung disease, use of cocaine, ecstasy, LSD, or IV drugs within the last year or currently receiving rehabilitation treatment for substance abuse, history of or current seizure disorder or on medications for seizures, neurological, psychiatric, or medical illness or injury expected to interfere with cognitive function including but not limited to stroke, head injury, diagnosed Alzheimer’s disease, or brain cancer, current depression. The clinical and cognitive evaluation included medical history, HF evaluation, a comprehensive neuropsychological evaluation, and a psychiatric rating scale for depression.

Control subjects enrolled in the study were 50 years or older and had no history of HF. Control participants were screened for COVID-19, and control participants with a known history of COVID-19 were excluded from the study. Global and domain-specific cognitive tests and blood collections were performed in both groups (control, and HF). For biomarker analyses, HF samples were compared with (i) prospectively collected control samples and (ii) age-matched retrospective control samples from the Precision Medicine True Normal Biobank.

HF participants were stratified based on HF progression using N-terminal pro-B-type natriuretic peptide (NT-proBNP), a biomarker indicative of HF severity and progression, used to guide clinical management. NT-proBNP levels, New York Heart Association (NHYA) status, ejection fraction (EF), medication relating to heart failure, comorbidities, and kidney function were obtained from the patient’s clinical history. The Estimate glomerular filtration rate (eGFR) was calculated from blood creatinine (Cr) levels using the CKD-EPI 2021 equation (MDCalc ©2005–2024)(Levey et al. 2009).

### Cognitive tests

Trained evaluators performed neuropsychological assessments. Neuropsychological assessments were given to HF and healthy control participants in separate rooms designed for cognitive assessment. Control (*n* = 11; average age: 63.20 years ± 7.86, 20.00% male) and HF (*n* = 34; average age: 68.62 ± 8.93, 61.76% male) participants were age-matched within the decade, with the greatest age difference being 7 years between individuals with HF and control subjects. The neuropsychological assessments contain a battery of tests for both global cognitive functional and domain-specific cognitive evaluations. The Montreal Cognitive Assessment (MoCA) (Nasreddine et al. 2005; Rossetti et al. 2011; Malek-Ahmadi and Nikkhahmanesh 2024) was used to evaluate global cognitive functional status, and the North American Adult Reading Test (NAART, CamCOPS NART 10.108) (Fromm et al. 1991; Bolla et al. 1998; Bright et al. 2018) was used to measure premorbid intellectual function. Additional cognitive domains assessed included (1) memory (verbal/visual associative memory, recognition, pattern separation), (2) executive functions (updating/working memory, inhibition, switching), and (3) processing speed (simple, complex). The complete list of cognitive tests is detailed in Table 1. These include Face-Name Associative Memory Exam (Amariglio et al. 2012; Papp et al. 2014; Fox et al. 2022), Verbal Paired Associates (VPA) (Talboom et al. 2019), Rey Auditory Verbal Learning Test (AVLT) (Geffen et al. 1994; Bean 2011), Mnemonic Similarity Task (MST) (Stark et al. 2013), Blobs (Ryan et al. 2012), Flanker (Eriksen and Eriksen 1974), Number-Letter (Rogers and Monsell 1995; Glisky et al. 2020), Keep Track (Glisky et al. 2020), and Deary Simple + Complex Reaction Time (Deary et al. 2011). Further explanation of the cognitive tests, their components, and *z*-score transformations can be found in the supplemental methods. Neuropsychological assessments were given to the same HF participants 12 months after their initial study (n is lower than the baseline as participant retention was low).

The Actual Composite Cognitive Score was constructed by selecting cognitive tests on which individuals with HF showed significant impairment compared to healthy control individuals. These included Verbal Paired Associates (VPA), Keep Track, and Face-Name tasks- cued name retrieval (CRN), cued occupation retrieval (CRO), and their 30-minute delayed versions (CRN30, CRO30). All test scores were standardized (z-scores) using the mean and standard deviation from the healthy control group. Equation (1) details the test components used for the Actual Composite Cognitive Scores.


(1) Actual Composite cognitive Score:CRN+CRN30+CRO+CRO30+VPA+KeepTrack

### Blood biomarkers

Blood samples were obtained after neuropsychological evaluations in the same HF participants detailed above. Blood samples were drawn for both plasma and serum. Blood designated for serum samples was collected in rapid serum tubes and exposed to room temperature for 30 minutes and then centrifuged for 10 minutes at 2000 × g. Blood collected for plasma was collected in lavender EDTA-coated blood tubes. Blood collected for plasma was kept on ice and then centrifuged for 15 minutes at 2000 × g. Serum and plasma samples were aliquoted into cryotubes and stored at −80 °C until analysis.

Neurodegenerative biomarkers assessment for HF (*n* = 24; average age: 68.09 ± 8.10; 60.87% male) participants were compared to age-matched healthy control samples (NFL: *n* = 20; average age: 64.10 ± 9.39, 40.00% male. pTau181: *n* = 13; average age: 68.85 ± 7.46, 38.46% male). Levels of serum neurofilament light protein (NfL) and phosphorylated-Tau181 (p-Tau181) were measured by PBLAssay Science (https://www.pblassaysci.com) using the Quanterix-Simoa assay (Quanterix #104073) and via the Simoa Human pTau181 V2 kit (MSD # K151AGMS-1). Serum placental growth factor (PlGF) was later run after NfL, and pTau181, by PBLAssay Science on a V-PLEX Angiogenesis Panel 1 Human Kit (K15190D). Samples used in the PlGF assay included both new and the same HF participants (*n* = 24) ran in NfL assays and were compared to age-matched healthy control participants (*n* = 24; average age: 65.21 ± 8.75; 45.83% males). For each serum assay, all samples were run in duplicate. Replicates across plates were averaged.

HF participants’ (*n* = 28) plasma pTau217 levels were measured later in the study using the Elecsys e801 from Roche Diagnostics in singlet at the Michael T. Zuendel Family Biomarker Laboratory, Banner Sun Health Research Institute.

Levels of serum inflammatory cytokines were determined using a multiplex assay (Millliplex: HCYTA-0N03231, Human Cyto/Chem/GF Panel A) with a MAGPIX Multiplexing Instrument and accompanying Multiplex Analyst software. The following analyte were measured simultaneously with the multiplex assay: Eotaxin, Fibroblast growth factor 2 (Fgf2), Interferon-gamma (IFNγ), interleukin (IL)-1a, IL-1b, IL-2, IL-4, IL-5, IL-6, IL-7, IL-8, IL-10, IL-12p40, IL-13, IL-15, IL-17a, IP-10, Macrophage-derived chemokine (MDC), macrophage inflammatory protein (MCP)-1, MIP-1α (CCL3), MIP-1b (CCL4), tumor necrosis factor-alpha (TNFα), tumor necrosis factor-beta (TNFβ), Vascular endothelial growth factor A (VEGFa). HF (*n* = 27; average age: 67.63 ± 8.77; 62.96% male) participant samples were compared with samples from healthy control (*n* = 25; average age: 65.77 ± 9.10; 50.00% male) individuals. All samples were run in duplicate. Values from multiple plates were normalized by repeated sample values run across plates (at least 3 or more samples were carried across all plates for normalization).

NT-proBNP levels of HF (*n* = 25) patients were provided from patient clinical histories. NT-proBNP levels were measured between 0 and 6 months prior to cognitive tests in clinically stable HF patients and were included for reference to represent chronic HF severity. In patients with stable HF, NT-proBNP demonstrates biological and clinical stability over several months, with little intra-individual variability except during acute decompensation or clinical events (Latini et al. 2004; Masson et al. 2006). Some participants were missing NT-proBNP levels; these individuals were excluded when stratified by NT-proBNP.

### Composite Biomarker Cognitive Score

To assess whether a combination of biomarkers could predict cognitive impairment, we generated a ‘Composite Biomarker Cognitive Score’ by the arithmetic combination of NfL, NT-proBNP, IL-6, and TNFα values. Equation (2) describes our method of calculating the Composite Biomarker Cognitive Score. All variables’ raw measured scores were included in the Composite Biomarker Cognitive Score and were transformed into a 1–4 scale and assigned values ranking 1–4 based on a range from low (1) to high (4) (Table 2).


composite Biomarker Cognitive Score=NfL+NT-proBNP+IL-6+TNFα

The inclusion criteria for neurodegenerative biomarkers for the Composite Biomarker Cognitive Score were that the neurodegenerative biomarkers had to show a significant increase in HF participants compared to age-matched healthy controls and significantly correlate with the Actual Composite Cognitive Score from Equation (1). Only NfL matched these criteria for neurodegenerative blood biomarkers to be included in the Composite Biomarker Cognitive Score. To transform NfL measured raw values, we established scores for NfL measured levels from published age- and disease-specific reference values (Vermunt et al. 2022). From these reference values, participants ≤50 years of age with a raw NfL (pg/mL) of ≤7pg/mL (50th percentile of healthy control values at 50 years) received an NfL score of 1. Participants ≤50 years with NfL values that are >7 to <11pg/mL (75th percentile for healthy controls at 50 years) received a NfL score of 2, and those with scores ≥11pg/mL (90th percentile for healthy controls at 50 years) received a NfL score of 4. Similar calculated scores were made for participants aged between 51 and 74 years of age, and for participants ≥75 years of age (Table 2). Reference NfL values for ages 51–74 were from published control individuals ages 55–70 (Vermunt et al. 2022). Reference values for participants ≥75 years of age were taken from published control individuals 75 years of age (Vermunt et al. 2022).

The inclusion criteria for blood-based cytokines in the Composite Biomarker Cognitive Score were that only serum cytokines that were significantly different in HF participants compared to healthy control samples and significantly correlated with the Actual Composite Cognitive Score were included. IL-6 and TNFα both met these criteria.

We established scores for IL-6 measured levels from published reference values in the Whitehall II longitudinal cohort study, which measured IL-6 levels from 7666 participants to predict cognitive decline (Singh-Manoux et al. 2014). From these referenced values, reported IL-6 values (pg/mL) ≥1.18 pg/mL (the lowest tertile of the referenced IL-6 values) received a score of 1. Those with values between 1.18 and 1.74 pg/mL (the middle tertile of the referenced IL-6 values) received a score of 2, and those with values >1.75 pg/mL (the highest tertile of the IL-6 referenced values) received a score of 4 (Table 2).

We established scores for TNFα measured levels from both published recent reference values (Serafini et al. 2024) and our own age-matched health control values. The mean±SD, min, and max TNFα levels pg/mL measured in our age and sex-matched healthy control values (*n* = 11) were 52.5 ± 14.1, 31.5, 81.6. Those with a TNFα value <80 pg/mL received a TNFα score of 1. Those with a TNFα value that was between 80 and 120 (3 standard deviations above the control mean) received a score of 2; those with values greater than 120 received a TNFα score of 4 (Table 2).

We established scores for NT-proBNP measured levels from published age, sex and disease-risk factor reference values in a large general population cohort (Welsh et al. 2022). From these reference values NT-proBNP values (pg/mL) <125 pg/mL received a NT-proBNP value of 1. Those with an NT-proBNP value between 125 and 300 pg/mL received a score of 2, and those with an NT-proBNP value >300 pg/mL received a score of 4 (Table 2).

### Statistical analysis

All data sets were tested for normality via the D’Agostino & Pearson test or the Shapiro-Wilk test if n was too low. Data sets that were normally distributed were tested for significance via Student’s T-test for two groups or an ANOVA for multiple variable groups, followed by a Tukey’s multiple comparisons test. For data sets that were not normally distributed, significance was tested using a Student Mann-Whitney test for two groups or a Kruskal-Wallis test followed by Dunn’s multiple comparisons test for multiple variable groups. For paired analysis comparing baseline to 12 months, the Student’s paired test was used if values met normality, or the Wilcoxon matched-pairs signed rank test if values did not meet normality. For all tests, a value of *p* ≤ 0.05 was considered significant. Of the biomarkers that exhibited significant differences between HF participants and controls, a linear regression model was used to examine the relationships between cognitive function scores and the significant biomarkers. All statistics were performed using GraphPad Prism10.1.0.

## Results

### Demographics and characteristics

Demographics, comorbidities, heart-based measurements, and medications for HF participants are listed in Table 3. Table 3 is separated into three groups: one control and two HF groups. Individuals with HF were stratified into two groups by NT-proBNP levels, ranging from 0–499 pg/mL, and ≥500 pg/mL.

HF individuals’ ages range from 50 to 85 years with an average age of 68.62 ± 8.93, *n* = 34. When stratified by NT-proBNP, the average age for HF patients with NT-proBNP ranging from 0–499 pg/mL was 67.71 ± 10.35 *n* = 14, and the average age for HF individuals with NT-proBNP levels greater than 500 pg/mL was 67.64 ± 9.52 *n* = 11(Table 3, *p* = 0.40 ANOVA). Healthy control individuals’ ages ranged from 50 to 81 years with an average of 64.41 ± 8.36, *n* = 34. Of the HF participants, 61.8% were male, and 45.5% were female in healthy control participants (HF vs. control *p* = 0.05 via Mann-Witney test). Corresponding age and sex are presented with the corresponding data sets.

Education across all participants was not significantly different when comparing HF participants, 15.29 ± 2.25, *n* = 34, to control, 16.46 ± 1.68, *n* = 11, (*p* = 0.13 via Mann-Whitney test).

Comorbidities in Table 3 are described as the percentage of individuals within each NT-proBNP group. Diabetes, chronic kidney disease (CKD), atrial fibrillation/flutter, and systemic hypertension are observed in over half of HF individuals with NT-proBNP greater than 500 pg/mL. Diabetes was significantly higher in HF patients with NT-proBNP levels ≥ 500 pg/mL compared to those with NT-proBNP levels of 499 pg/mL or less (*p*=0.03 Mann-Whitney test, Table 3). Coronary artery disease (CAD) and systemic hypertension (HNT) were observed in 50% or more patients with NT-proBNP ranging from 0 to 499pg/mL. Excluding diabetes, there were no significant differences in comorbidities between HF groups stratified by NT-proBNP. Similarly, there were no significant differences in Creatinine, BUN, or eGFR between HF groups stratified by NT-proBNP (Creatinine: *p* = 0.08, BUN: *p* = 0.95, eGFR: *p* = 0.29 via student t-test, Table 3).

The average ejection fraction (EF) for all HF participants is 37.11%, *n* = 31. HF patients with NT-proBNP levels <500 pg/mL, had an EF of 40.25 ± 13.27 *n* = 14 that was significantly higher than patients with an NT-proBNP level of 500 pg/mL or higher, 24.78 ± 8.42 *n* = 9 (Table 3, *p* = 0.005 Student’s T-test). Those with NT-proBNP levels <499 pg/mL had HF symptoms that were classified within the range of II-III on the New York Heart Association (NYHA) classification, and those with NT-proBNP levels greater than 500 pg/mL had symptoms that classified their HF as NYHA II-IV. These metrics indicate that stratification with NT-proBNP in our patient population reflects potentially worsened heart failure, not only by biomarker levels of NT-proBNP itself but also by heart function via EF and NYHA classification.

Medications are listed as a percentage of individuals for each medication class. Under each percentage is a list of the generic medications taken.

### Individuals with heart failure have cognitive impairment

HF participants demonstrate significantly lower cognitive performance than healthy control participants (Table 4). Global cognitive function, as measured by the MoCA, was lower in the HF participants compared to control participants (Control MoCA z-score: 0.93 ± 0.73, *n* = 10; HF MoCA z-score: −0.44 ± 1.38, *n* = 32; *p* value = 0.005 via student t-test). HF participants scored lower on the NAART compared to controls (Control NAART Total Correct: 49.00 ± 5.81, *n* = 10; HF NAART Total Correct: 37.79 ± 8.80, *n* = 33; *p* value = 0.0001 via Mann-Whitney test). In Addition, HF participants performed significantly worse on specific domain tasks, including, Face Name, VPA, Keep Track, MST, Number Letter, Flanker, Deary, and Blobs (Table 4, Supplemental Table 1).

**Figure 1.**
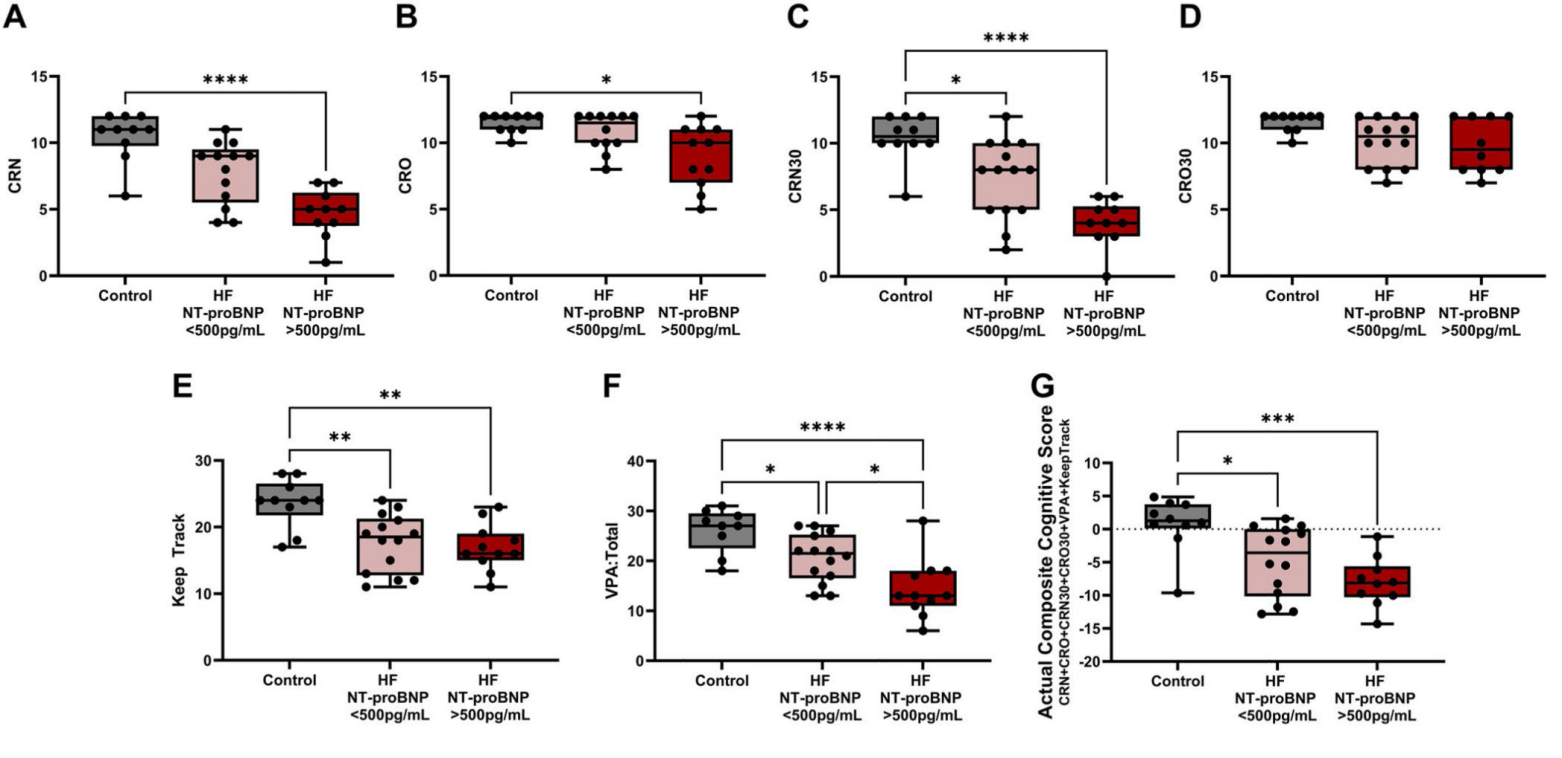
Cognitive performance appears to decrease with worsened HF status. Cognitive values for heart failure (HF) participants were stratified by N-terminal pro-B-type natriuretic peptide (NT-proBNP) levels and compared to healthy control participants (A) cued name retrieval (CRN), (B) cued object retrieval (CRO), (C) 30-minute delay cued name retrieval (CRN30), (D) 30-minute delay cued occupation retrieval (CRO30), (E) Keep Track, and (F) Verbal Paired associated (VPA). CRN, CRO, CRN30, and CRO30 are all tasks part of FNAME. (G) A composite score (Actual Composite cognitive Score) was made using z-transformed values of CRN, CRO, CRN30, CRO30, keep track, and VPA. Groups were tested for normality via d’Agostino & Pearson test or Shapiro-Wilk test if *n* was too low. Multiple variables meeting normality were tested for significance via ordinary one-way ANOVA, and comparisons between groups were tested for significance Tukey’s multiple comparisons test. If values do not meet normality, multiple variables were tested for significance via the Kruskal-Wallis test, and comparison between groups was tested for significance through Dunn’s multiple comparisons test. *p* value indicated by *. *p* = 0.05–0.01 *, *p* = 0.009–0.001 **, *p*=0.0009–0.0001 ***, *p*< 0.0001 ****.

HF participants performed significantly worse on certain memory-related tasks that test visual associative memory, verbal associative memory, and working memory, as HF patients performed worse on FNAME, VPA, and Keep Track tasks. In general, HF patients performed significantly worse in tasks of recalling names and occupations compared to healthy control individuals (Table 4). HF patients scored worse on CRN (cued name retrieval), CRO (cued occupation retrieval), CRN30 (30-minute delay cued name retrieval), CRO30 (30-minute delay cued occupation retrieval), MCN, FNN (total correct recall of names across learning trials, short, and long delay), FNO, and Total FNAME Score. Individuals with HF had difficulty in performing tasks that required name retrieval following either a short or long delay. When stratified by HF maker, NT-proBNP’s ability to recall names and occupations immediately after learning the subject appeared to decrease as the level of NT-proBNP increases (Figure 1A,B). CRN: Control 10.50 ± 1.84, *n* = 10; HF NT-proBNP < 500 pg/mL 7.77 ± 2.35, *n* = 13; HF NT-proBNP ≥500 pg/mL 4.70 ± 1.83, *n* = 10; CRO: Control 11.50 ± 0.71, *n* = 10; HF NT-proBNP < 500 pg/mL 10.83 ± 1.40, *n* = 12; HF NT-proBNP ≥ 500 pg/mL 9.00 ± 2.32, *n* = 11). Similarly, HF participants performed worse with name recall after a 30-minute delay as NT-proBNP levels increased (Figure 1C). Control 10.00 ± 1.78, *n* = 10; HF NT-proBNP < 500 pg/mL 7.36 ± 2.93, *n* = 14; HF NT-proBNP ≥500 pg/mL 4.00 ± 1.76, *n* = 10). HF participants also had significantly decreased performance on both Keep Track (Keep Track Total), and VPA (VPA: Total; Table 4). Similar to FNAME tasks, performance decreased with increased levels of NT-proBNP in Keep Track tasks (Figure 1E). Control 23.60 ± 3.66, *n* = 10; HF NT-proBNP < 500 pg/mL 17.64 ± 4.34, *n* = 14; HF NT-proBNP >500pg/mL 16.91 ± 3.53, *n* = 11) and significantly decreased with increased NT-proBNP levels in VPA tasks (Figure 1F). Control 26.11 ± 4.43, *n* = 9; HF NT-proBNP < 500 pg/mL 20.57 ± 4.83, *n* = 14; HF NT-proBNP >500 pg/mL 14.36 ± 5.84, *n* = 11).

The variables that are constituents for FNAME (CRN, CRO, CRN30, and CRO30), VPA, and Keep Track were z-score transformed and calculated into the ‘Actual Composite Cognitive Score’ (Table 4). HF participants had significantly poorer performance on the corresponding tests and, therefore, had lower composite scores compared to healthy control participants (Table 4, HF: −5.48 ± 4.84 *n* = 32, Control: 0.78 ± 4.10, *n* = 10, *p* = 0.0001 via Mann-Whitney test).

HF participants also performed worse on particular aspects of multiple other tasks, including MST, Number Letter, Flanker, Deary, and Blobs (Supplemental Table 1). This included misidentifying new objects for certain MST tasks, slower switching and increased reaction time on certain Number Letter tasks, decreased accuracy in Flanker tasks, and an overall decreased accuracy in the Blobs tasks.

### Individuals with HF have increased serum neurodegenerative biomarkers and cytokines

**Figure 2.**
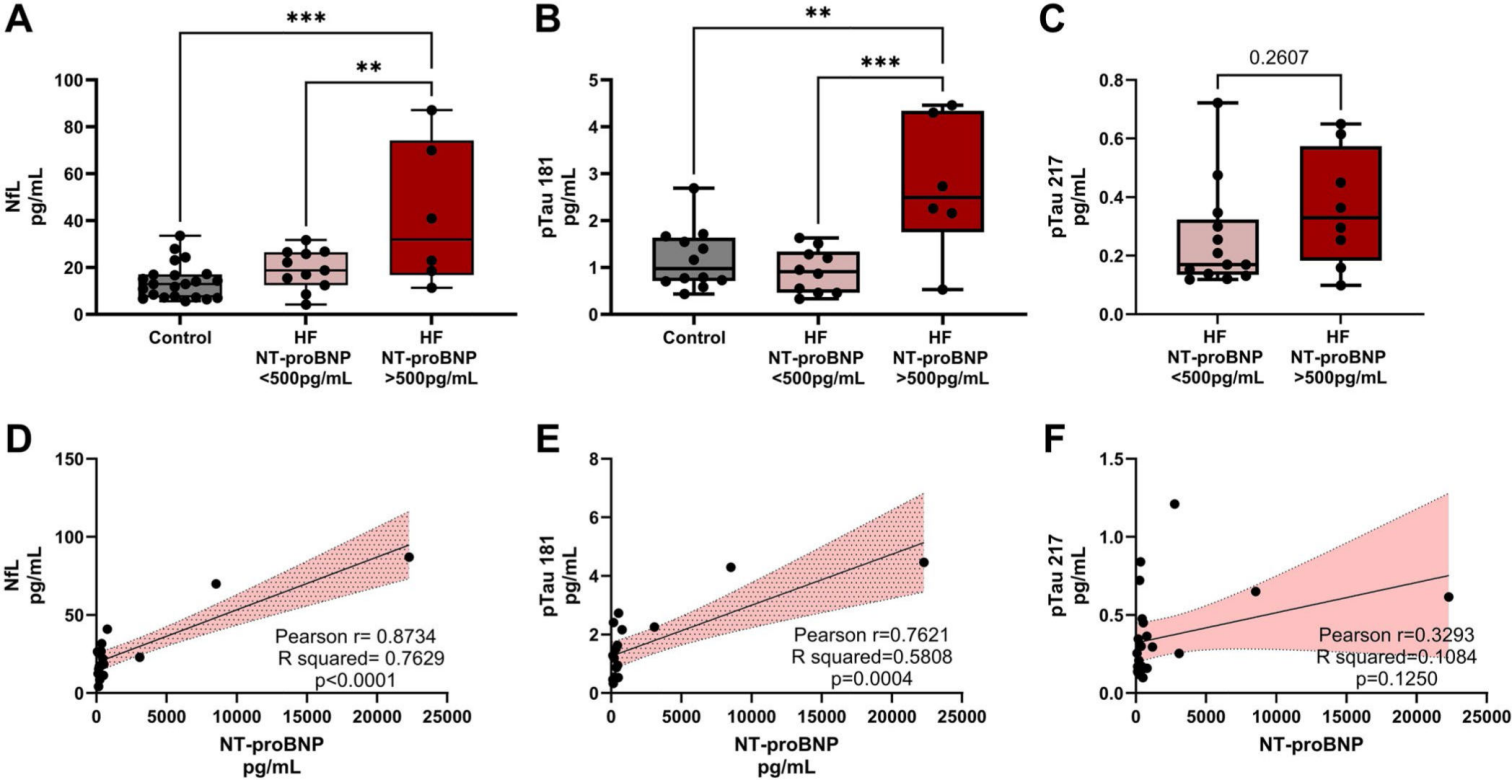
VCID neurodegenerative biomarkers appear to increase with worsened HF status, but not pTau217. Serum neurodegenerative values for heart failure (HF) participants were stratified by N-terminal pro-B-type natriuretic peptide (NT-proBNP) levels and compared to healthy control participants (A) neurofilament light protein (NfL), and (B) phosphorylated tau 181 (pTau181). (C) No significant differences were observed in plasma phosphorylated tau 217 (pTau217) levels in HF participants when stratified by NT-proBNP. Groups were tested for normality via d’Agostino & Pearson test or Shapiro-Wilk test if n was too low. Multiple variables were tested for significance via ordinary one-way ANOVA, and comparisons between groups were tested for significance using Tukey’s multiple comparisons test. (A, B) Significance was tested between the two variables using the Mann-Whitney test. (C) Correlation between NT-proBNP, a marker of heart failure severity, and (D) NfL, (E) pTau181, and (F) pTau217 were tested with a simple linear regression, interactions represented via R squared, and correlation is represented via Pearson *r* values. *p* value indicated by *. *p*=0.05–0.01 *, *p* = 0.009–0.001 **, *p* = 0.0009–0.0001 ***, *p* < 0.0001 ****.

Serum NfL levels were significantly higher in HF participants (18.84 pg/mL ± 8.76, *n* = 21) compared to age-matched healthy control participants (13.15 pg/mL ± 6.34, *n* = 21; *p* = 0.02 via student’s t-test) (Table 5). When stratified by NT-proBNP (an indicator of HF status), NfL increased with increased NT-proBNP values (Figure 2A). Control 14.02 pg/mL ± 7.57, *n* = 22; HF NT-proBNP < 500 pg/mL 19.06 pg/mL ± 8.51, *n* = 11; HF NT-proBNP ≥500 pg/mL 41.81 pg/mL ± 30.55, *n* = 6). Similarly, pTau181 also increases with increased NT-proBNP (Figure 2B). Control 1.18 pg/mL ± 0.65, *n* = 12; HF NT-proBNP < 500 pg/mL 0.92 pg/mL ± 0.47, *n* = 10; HF NT-proBNP ≥500 pg/mL 2.74 pg/mL ± 1.47, *n* = 6). Both NfL and pTau181 in participants who have NT-proBNP levels of 500 pg/mL or higher are significantly higher than the HF patients with lower NT-proBNP levels and the healthy control groups. We further show that these circulating neurodegenerative biomarkers significantly positively correlate with HF severity represented as NT-proBNP (Figure 2D). NfL: Pearson *r*=0.87, R squared = 0.76, *p* = <0.0001; Figure 2E. pTau181: Pearson *r*=0.76, R squared= 0.58, *p* = 0.0004; tested via simple linear regression). pTau217, an indicator of amylodopathies (Ashton et al. 2023; 2024), did not significantly increase across HF severity groups stratified by NT-proBNP, nor was it correlated with increasing NT-proBNP levels (Figure 2C,F). These results indicate that neurodegenerative biomarkers NfL, and pTau181 increase with worsening HF.

**Figure 3.**
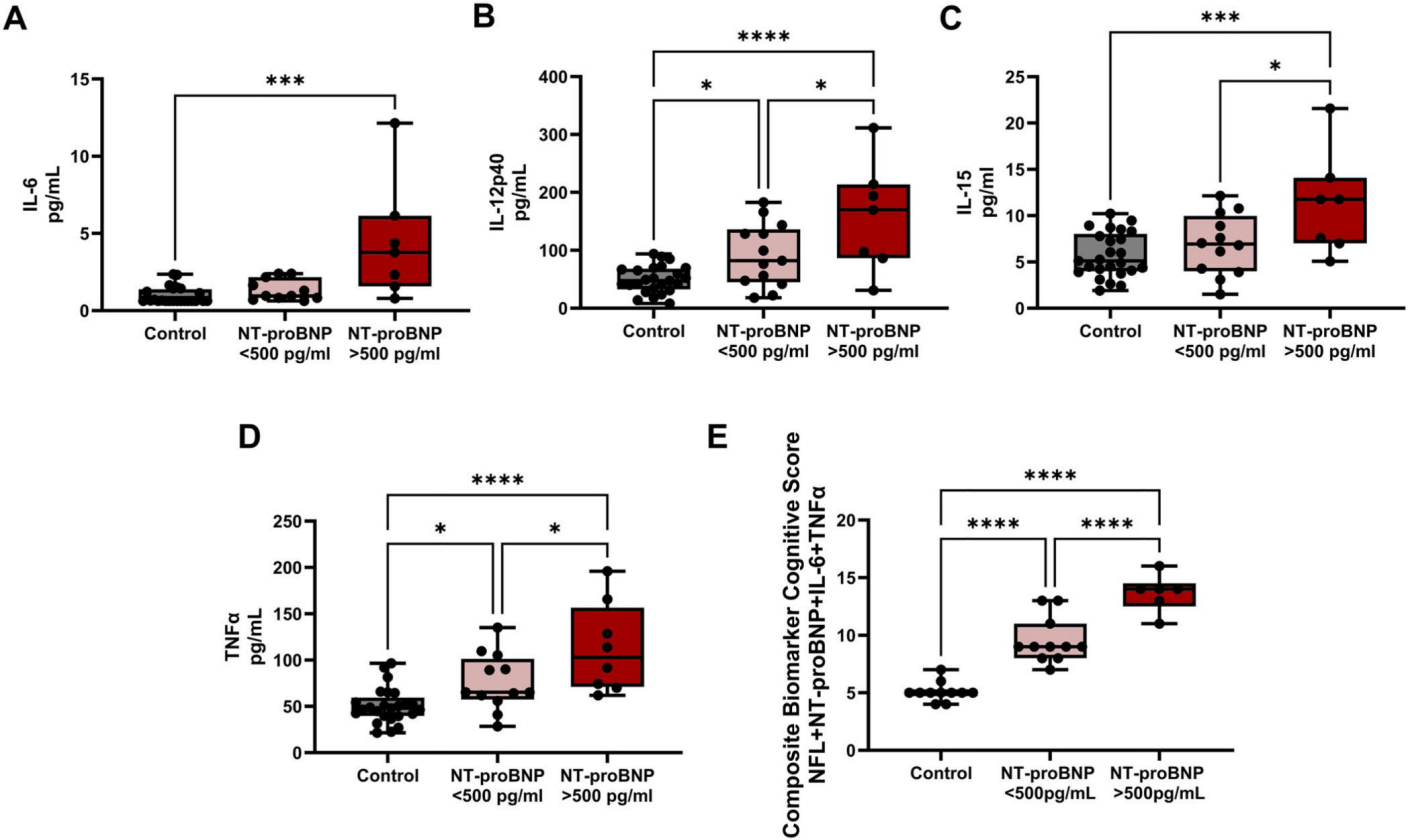
Serum cytokine values and the Composite Biomarker Cognitive Score increase with worsened HF status. Cytokine values for heart failure (HF) participants were stratified by N-terminal pro-B-type natriuretic peptide (NT-proBNP) levels and compared to healthy control participants (A) interleukin (IL)6, (B) IL-12p40, (C) IL-15, (D) and tumor necrosis factor-alpha (TNFα). (E) A Composite Biomarker Cognitive Score was made using transformed values of neurofilament light protein (NfL), IL-6, TNFα, and N-terminal pro-B-type natriuretic peptide (NT-proBNP). Groups were tested for normality via d’Agostino & Pearson test or Shapiro-Wilk test if n was too low. Multiple variables meeting normality were tested for significance via ordinary one-way ANOVA, and comparisons between groups were tested for significance Tukey’s multiple comparisons test. If values do not meet normality, multiple variables were tested for significance via the Kruskal-Wallis test, and comparison between groups was tested for significance through Dunn’s multiple comparisons test. *p* value indicated by *. *p* = 0.05–0.01 *, *p* = 0.009–0.001 **, *p*=0.0009–0.0001 ***, *p* < 0.0001 ****.

Serum levels of IL-6, IL-12p40, IL-15, MIP-1α, TNFα, and TNFβ were significantly increased in HF participants compared to control participants (Table 5; IL-6 *p* < 0.0001 Mann-Whitney test, IL-12p40 *p*< 0.0001 students t-test, IL-15 *p* = 0.005 students t-test, MIP-1α *p* = 0.007 Mann-Whitney test, TNFα *p* = 0.0002 students t-test, and TNFβ *p* = 0.03 Mann-Whitney test). IL-6, IL-12p40, IL-15, and TNFα increase as NT-proBNP increases (Figure 3). Cytokine levels were significantly higher in HF participants with NT-proBNP levels of ≥500pg/mL compared to HF individuals with lower NT-proBNP levels for IL-12p40, IL-15, and TNFα (Figure 3B-D).

### Blood biomarkers correlate to cognitive performance

Of the circulating biomarkers that were significantly higher in HF compared to healthy control participants, NfL, IL-6, and TNFα significantly correlated with the Actual Composite Cognitive Score calculated from FNAME, VPA, and Keep Track. With these biomarkers and NT-proBNP, we created a Composite Biomarker Cognitive Score (Equation (2)). The Composite Biomarker Cognitive Score combines neurodegenerative serum biomarkers, NfL, proinflammatory serum cytokine, IL-6, and TNFα, and an HF biomarker NT-proBNP to predict an individual’s cognitive performance in HF individuals. HF participants’ Composite Biomarker Cognitive Score (10.00 ± 3.09, *n* = 23) was significantly higher compared to healthy control participants (5.09 ± 0.83, *n* = 11, *p* < 0.0001, Student’s t-test (Table 5).

**Figure 4.**
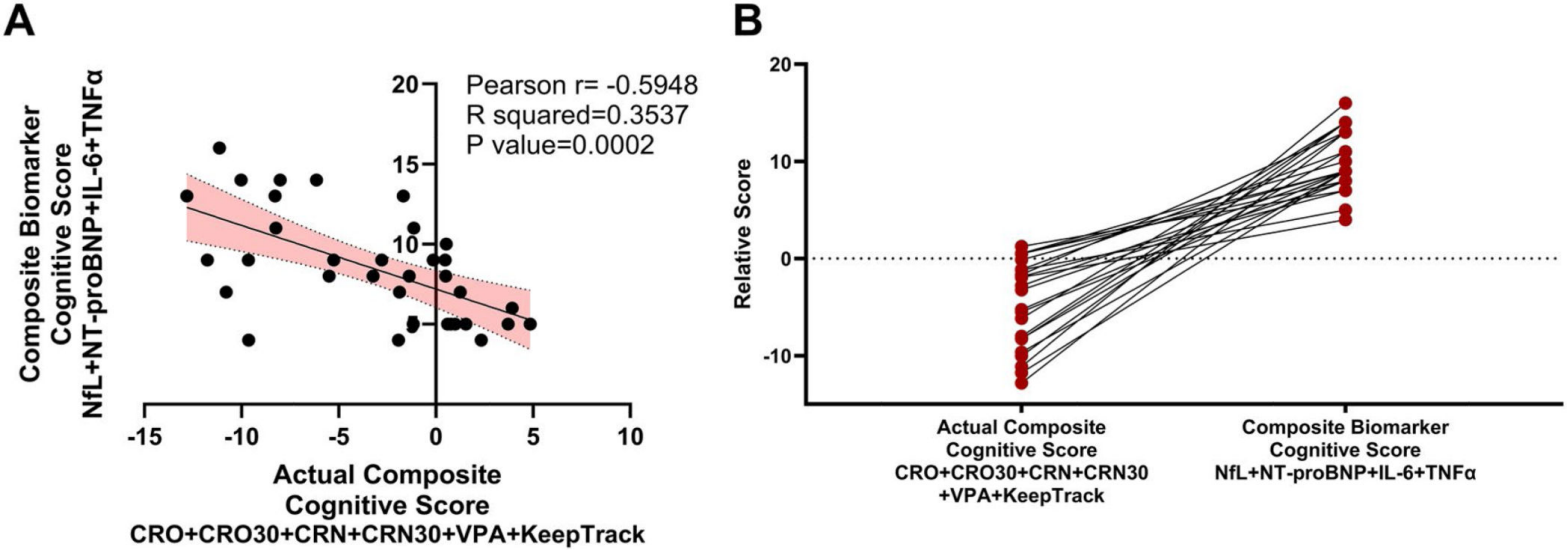
The Composite Biomarker Cognitive Score correlated to the actual Composite cognitive Score. (A) Correlation and interaction between the Composite Biomarker Cognitive Score and the actual Composite cognitive Score were tested via simple linear regression, interactions represented via R-squared, and correlation is represented via Pearson *r* values. (B) Visualization of the Composite Biomarker Cognitive Score to predict the actual Composite cognitive Score is demonstrated in the dot line graph (measured in relative score). The Composite Biomarker Cognitive Score = NfL+NT-proBNP + IL-6 + TNFα. The actual Composite cognitive Score = CRN + CRN30 + CRO + CRO30 + VPA + KeepTrack.

In order to establish the potential for the Composite Biomarker Cognitive Score to predict cognitive impairment in HF patients we correlated the Composite Biomarker Cognitive Score to the Actual Composite Cognitive Score. The Composite Biomarker Cognitive Score is significantly negatively correlated to the Actual Composite Cognitive Score (Figure 4, Table 6, Pearson *r* = −0.59, *p* = 0.0002), such that a higher Composite Biomarker Cognitive Score was related to poorer cognitive performance. As the Composite Biomarker Cognitive Score increased, reflecting an increase in neurodegeneration, inflammation, and/or heart failure, the Actual Composite Cognitive Score decreased, reflecting a decline in performance on the FNAME, VPA, or Keep Track neurocognitive tasks. In addition, the Composite Biomarker Cognitive Score significantly correlated with each individual measure of the FNAME and with the total VPA and Keep Track scores, further supporting the Composite Biomarker Cognitive Score to predict cognitive performance (Table 6). These results suggest that the Composite Biomarker Cognitive Score could potentially predict HF participants’ cognitive impairment.

### Longitudinal data show that decreases in cognitive performance are observed, accompanied by increases in circulating cytokines

We were able to obtain longitudinal data from 6 HF participants to identify possible changes in cognitive performance and serum cytokine levels over 12 months. Blood and neurocognitive assessments were successfully collected from 5 of 6 participants.

**Figure 5.**
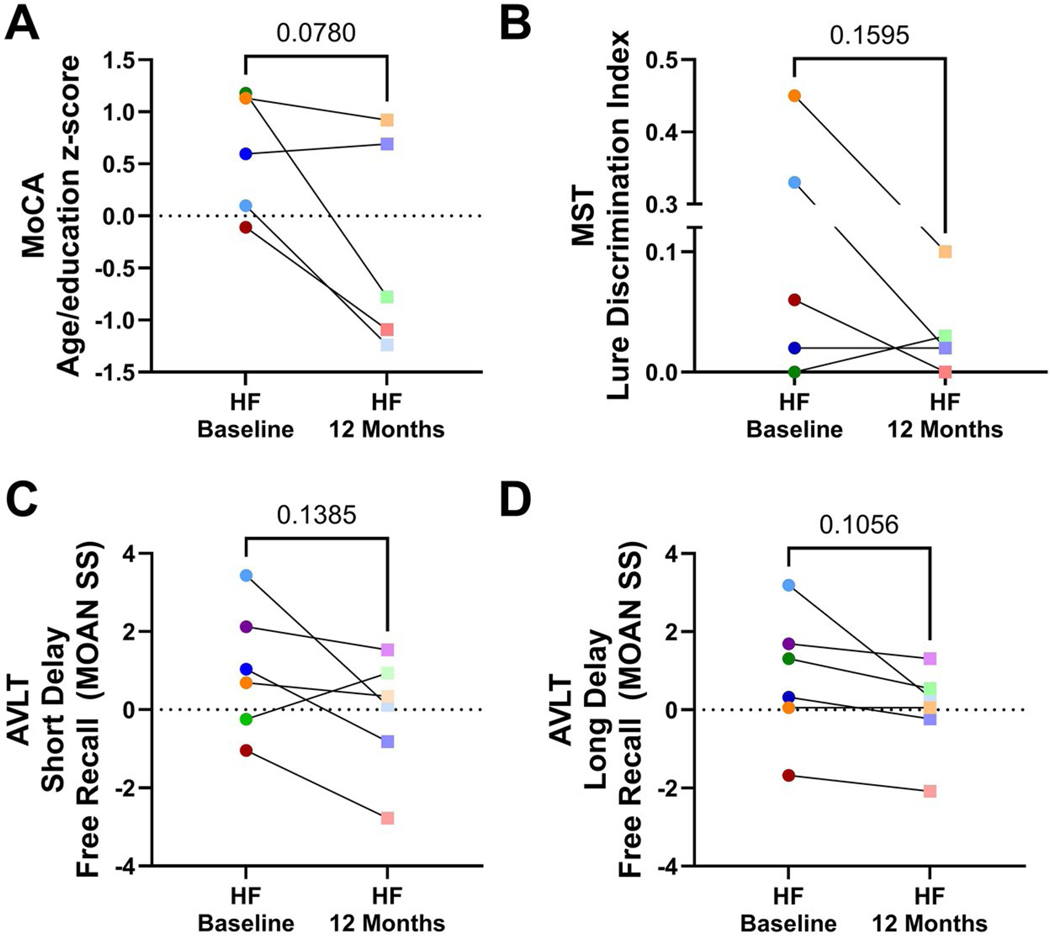
HF participants performed worse on cognitive and functional tests 12 months after their baseline visit. Values from baseline neuropsychological evaluations were compared to 12-month follow-up values and represented in the dot line graphs, showing the progression of each individual over time from baseline to 12-month follow-up for (A) the age and education normalized score from the Montreal cognitive assessment (MoCA), (B) the lure discrimination index from the mnemonic similarity task (MST), and both (C) the short delay free recall (MOAN standard Score) and D) the long delay free recall (MOAN standard Score) tasks from the rey auditory Verbal Learning test (AVLT). Individual participant’s colors are assigned for each person. Round, more saturated colors indicate individuals’ scores at baseline, and the less saturated square indicates 12-month follow-up values from the corresponding individuals. Normality was tested using the Shapiro-Wilk test. Significance between baseline and 12-month follow-up was tested for significance via Student’s paired test.

The longitudinal data from this small subgroup of our population suggest a decrease in cognitive performance over 12 months from baseline tests in the MoCA, Lure discrimination index (MST), and Short Delay Free Recall, and Long Delay Free Recall tasks of the AVLT (Figure 5). 4 out of 5 individuals performed worse in their average MoCA (age and education transformed) score after 12 months (Figure 5A); on average HF participants MoCA scores decreased from 0.58 ± 0.59 at baseline to −0.30 ± 1.03 (Figure 5A, Table 7). We saw similar trends with the Lure Discrimination Index task of the MST (Figure 5B and Table 7), and in both the Short Delay Free Recall, and Long Delay Free Recall tasks of the AVLT (Figure 5C,D and Table 7).

**Figure 6.**
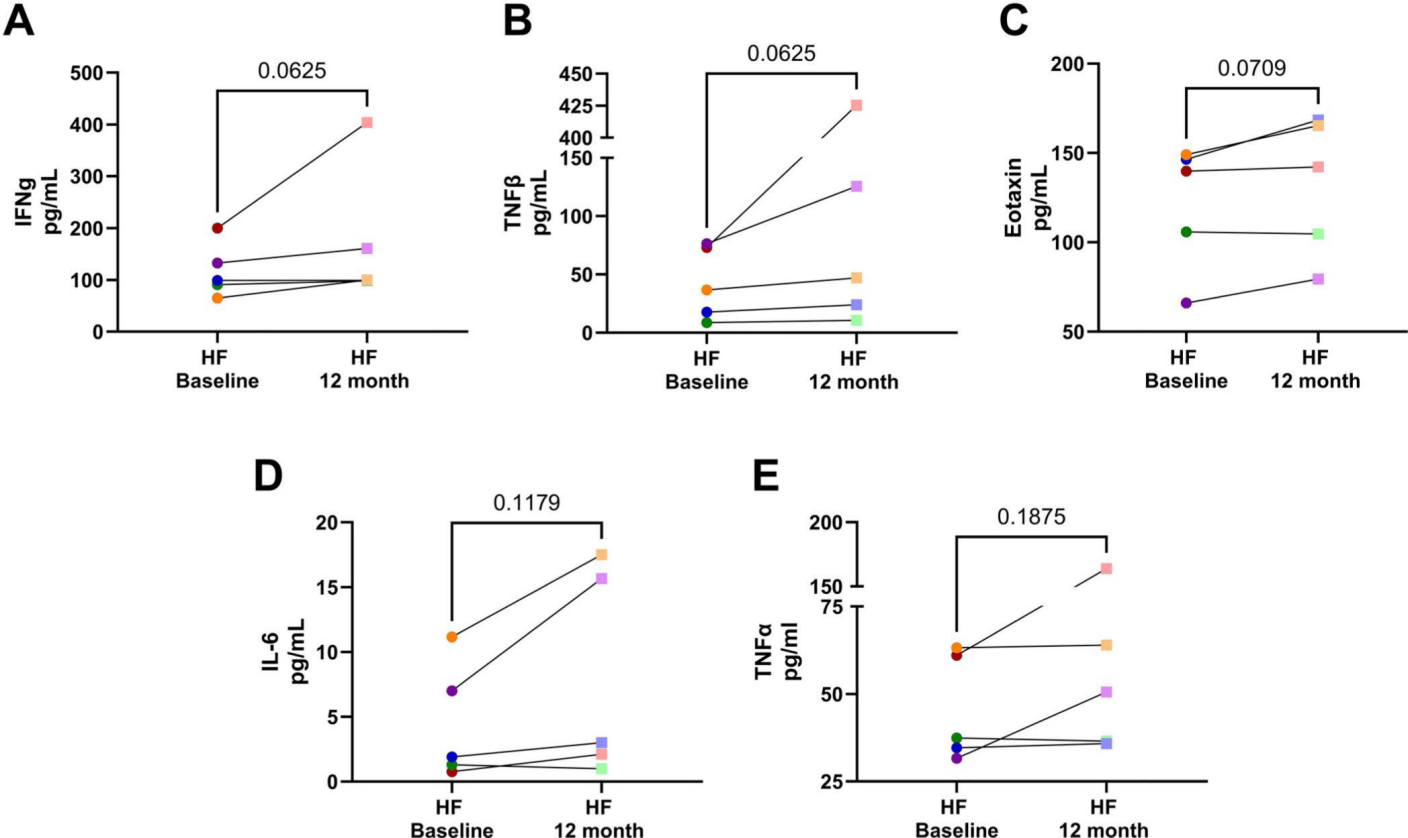
HF participants had increased serum cytokine 12 months after their baseline visit. Values of baseline serum cytokine were compared to 12-month follow-up values and represented in the dot line graphs, showing the progression of cytokine values for each individual over time from baseline to 12-month follow-up for (A) Interferon-gamma (INFγ), (B) tumor necrosis factor-beta (TNFβ), (C) Eotaxin, (D) interleukin (IL)-6, and (E) tumor necrosis factor-alpha (TNFα). Individual participants’ colors are assigned for each person. Round, more saturated colors indicate individuals’ scores at baseline, and the less saturated square indicates 12-month follow-up values from the corresponding individuals. Normality was tested using the Shapiro-Wilk test. Significance between baseline and 12-month follow-up was tested via Student’s paired test if values met normality (Eotaxin, and IL-6), or Wilcoxon matched-pairs signed rank test if values did not meet normality (INFγ, TNFβ, and TNFα).

Cytokine values for participants in the longitudinal arm of our study showed an increase over 12 months from baseline for IFNγ for 5 of 5 participants, TNFβ for 5 of 5 participants, Eotaxin for 4 of 5 participants, IL-6 for 4 of 5 participants, and TNFα for 4 of 5 participants, (Figure 6A-E); and the average score of all 5 HF participants increased from baseline to the 12-month follow-up (IFNγ Baseline: 117.43 ± 52.05, 12 Month follow-up: 172.22 ± 132.24; TNFβ Baseline: 42.55 ± 31.09, 12 Month follow-up: 126.54 ± 172.88; Eotaxin Baseline: 121.41 ± 35.48, 12 Month follow-up: 131.98 ± 38.87; IL-6 Baseline: 4.43 ± 4.52, 12 Month follow-up: 7.85 ± 8.02; and TNFα Baseline: 45.57 ± 15.28, 12 Month follow-up: 70.15 ± 53.66 (Figure 6 and Table 8).

## Discussion

Results from this study are the first to create a Composite Biomarker Cognitive Score, which is highly predictive of cognitive impairment in HF individuals. Our Composite Biomarker Cognitive Score combines neurodegenerative serum biomarkers, NfL, proinflammatory serum cytokine, IL-6, and TNFα, and HF biomarker NT-proBNP to predict an individual’s cognitive performance in HF individuals (Composite Biomarker Cognitive Score = NfL + NT-proBNP + IL-6 + TNFα). The Composite Biomarker Cognitive Score was significantly correlated with the Actual Composite Cognitive Scores. These findings suggest that cognitive impairment in HF may be predicted using a combination of neurodegeneration, inflammatory, and cardiac function biomarkers and may be useful in identifying individuals who could benefit from future cognitive protective therapies that are currently under development.

We further showed that cognitive abilities (especially reflected via the MoCA) may further decrease in HF participants over time and that these impairments are accompanied by an increase in serum cytokines, thus potentially predicting longitudinal cognitive decline in HF patients.

### Cognitive impairment in heart failure

With 40%−60% of individuals with heart failure (HF) experiencing cognitive impairment and being at risk for VCID, there is a need for a biomarker(s) that can detect neuronal damage in at-risk individuals before clinical cognitive dysfunction develops, enabling early intervention (Vogels et al. 2007). Our results confirm previous studies’ findings that up to 20%−80% of individuals with HF experience cognitive impairment (Redwine et al. 2018; Yang et al. 2021; Goh et al. 2022; Traub et al. 2022b). In the present study, we demonstrate that HF participants showed poorer cognitive performance on MoCA and on domain-specific tasks (associative memory and working memory), consistent with prior reports. NAART was collected to estimate premorbid verbal/reading ability and is presented for context rather than as evidence of HF-related cognitive decline. HF patients also performed significantly worse on specific components across cognitive tests for domains in verbal/visual associative memory, working memory, pattern separation, switching, inhibition, and object discrimination. Cognitive assessments of individuals with HF typically are based on Mini-Mental State Examination (MMSE) (Vogels et al. 2007), the MoCA (Leto and Feola 2014; Celutkiene et al. 2016), or Trail-Making Task (TMT) (Vogels et al. 2007; Leto and Feola 2014). Similar to our results, in other studies, individuals with HF perform worse than healthy control individuals in functional and cognitive tests (Heckman et al. 2007). In a study by Athilingam et al., 69 participants with systolic heart failure mean MoCA score was 22.90 ± 2.31, and 24.80 ± 2.76 for individuals with diastolic heart failure (*n* = 21), comparable to our average score for HF (24.29 ± 3.01) (Athilingam et al. 2013a). While less frequently used in HF cognitive assessments, the NAART has been shown to be impaired in dementia and correlated with the severity of dementia in Alzheimer’s disease (Fromm et al. 1991). Previous studies have also shown the NAART to estimate premorbid verbal abilities of individuals with mild to moderate dementia (Fromm et al. 1991).

Within our study, HF participants also performed significantly worse on certain memory-related tasks that test visual, associative memory, verbal associative memory, and working memory, as HF patients performed worse on FNAME, VPA, and Keep Track tasks (Papp et al. 2014; Talboom et al. 2019; Glisky et al. 2020). Other studies have also shown that individuals with HF have significant impairment in working memory, immediate memory, and delayed memory (Bauer et al. 2011). In a study by Trojano et al. with 149 moderate HF cases and 159 severe heart failure cases, they showed that HF participants scored significantly lower on verbal memory tasks measured by AVLT (Trojano et al. 2003). While AVLT was not significantly decreased in our HF participants at baseline, during our longitudinal pilot study, the immediate recall (trial 1 of list A) showed a significant decrease in HF participants 12 months after baseline compared to healthy controls.

### Serum biomarkers for neurodegeneration and cytokine levels are increased in participants with heart failure

To identify biomarkers specific to HF and cognitive impairment, we showed that serum biomarkers for neurodegenerative disease, NfL, and cytokines, IL-6, IL-12p40, IL-15, MIP-1α, TNFα, and TNFβ, levels were significantly higher in HF participants compared to healthy control participants. Of the circulating biomarkers that were significantly higher in HF, NfL, IL-6, and TNFα significantly correlated with the Actual Composite Cognitive Score calculated from FNAME, VPA, and Keep Track tasks. While our study is the first to combine these biomarkers into a Composite Biomarker Cognitive Score, previous studies have also shown NfL to be increased in HF patients and correlated with cognitive impairment (Traub et al. 2022b). Traub et al. also showed a positive correlation between NfL and pTau181 vs hippocampal and cerebral atrophy, areas that are important for memory and executive function (Traub et al. 2022b). When stratified by NT-proBNP (an indicator of HF status), we observed that NfL increases with increased NT-proBNP values and is significantly positively correlated with NT-proBNP. These results support previous studies that indicate that as HF severity progresses, NfL levels increase (Traub et al. 2022b). Other studies have also observed that NfL levels of individuals with either HFrEF or HFpEF positively correlate with NT-proBNP (Lahiri et al. 2022), or creatine kinase levels (Traub et al. 2022a). These results indicate that NfL is a sensitive biomarker to cognitive decline in HF patients.

We also investigated other neurodegenerative biomarkers, including pTau181 and PlGF. In our study, pTau181 was significantly correlated to our Actual Composite Cognitive Score. Similar to our NfL results, pTau181 also increased when stratified by NT-proBNP and was significantly positively correlated with HF severity represented as NT-proBNP. These results indicate that the pTau181 increases with worsening HF. Studies performed by Traub et al. also showed a significant correlation between pTau181 and visual/verbal memory impairment and NT-proBNP (Traub et al. 2022b). Plasma PlGF levels have been shown to be correlated to cerebral small vessel disease, white matter hyperintensities, and VCID (Kern et al. 2025). However, we observed no significant changes or correlations in serum PlGF levels in our VCID at-risk HF population.

As inflammatory processes play an important role in HF and cognitive impairment, serum cytokine levels pose as potential biomarkers to identify cognitive impairment in HF (Athilingam et al. 2013b). In the present study, serum levels of IL-6, IL-12p40, IL-15, MIP-1α, TNFα, and TNFβ were significantly increased in HF participants compared to control participants. While IL-12p40, IL-15, and TNFα of HF participants with NT-proBNP ≥500 pg/mL were significantly higher than HF participants with NT-proBNP levels of <500 pg/mL, these cytokines do not significantly correlate with NT-proBNP levels, an indicator of heart failure progression. However, IL-6 and TNFα are significantly correlated with our Actual Composite Cognitive Score. These data suggest that increased levels of these circulating cytokines are closely able to reflect outcomes affecting cognitive impairment.

### Longitudinal decrease in cognitive performance is accompanied by increased cytokine levels

Our longitudinal data is from a small subset of participants and should be interpreted with caution. Data from 6 HF participants were gathered to identify possible changes in cognitive performance and serum cytokine levels over 12-month period. Blood and neurocognitive assessments were successfully collected from 5 of 6 participants. These longitudinal data may suggest a decrease in cognitive performance over 12 months from baseline tests in the MoCA, lure discrimination index (MST), Short Delay Free Recall, and Long Delay Free Recall tasks of the AVLT. We also investigated if cytokine values changed longitudinally in HF. Values for participants tended to increase over 12 months from baseline for IFNγ for all participants, TNFβ for all participants, TNFα for 4 of 5 participants, and IL-6 for 4 of 5 participants.

## Limitations and future directions

The major limitation of this manuscript is the limited sample size, which limits any evaluations of the effects of race/ethnicity, sex, or education level. In future studies, we plan on expanding our sample size to further examine the ability of blood-based biomarkers to predict cognitive function in the population of VCID-HF individuals. Moreover, larger, independent studies will be critical to replicate these findings and to further validate both the Actual Composite Cognitive Score and the Composite Biomarker Cognitive Score as reliable measures of cognitive risk in HF populations.

Further, we plan on additional longitudinal studies to show how our cognitive panel may predict cognitive impairment outcomes/trajectory, which are needed to establish our cognitive panel as a potential predictive biomarker for dementia and vascular cognitive impairment associated with HF. Lastly, we did not measure APOE status in our cohort.

## Conclusion

In this study, for the first time, we created a Composite Biomarker Cognitive Score, encompassing neurodegenerative serum biomarkers, NfL, proinflammatory serum cytokine, IL-6, and TNFα, and HF biomarker NT-proBNP to predict an individual’s cognitive performance in HF individuals. Our Composite Biomarker Cognitive Score was significantly correlated with Actual Composite Cognitive Scores. Our results indicate that cognitive impairment in HF may be predicted using a combination of neurodegeneration, inflammatory, and cardiac function biomarkers and may be useful in identifying individuals who could benefit from future cognitive protective therapies.

## Supplementary Material

Supp 1

## Figures and Tables

**Table 1.** A list of the cognitive tests included in the neuropsychological evaluation.

Test	Domain
Cognitive and Premorbid Function Tests	
Montreal Cognitive Assessment	Global cognitive function
North American Adult Reading Test (NAART)	Premorbid intellectual function, word reading
Domain-Specific Cognitive Test	
Face-Name Associative Memory Exam (FNAME)	Visual associative memory
Verbal Paired-Associates Learning (VPA)	Verbal associative memory
Rey Auditory Verbal Learning Test (AVLT)	Verbal memory, list learning
Mnemonic Similarity Task (MST)	Object recognition and pattern separation
Blobs	Object discrimination
Flanker	Attention/inhibition
Number-Letter	Switching
Keep Track	Updating/working memory
Deary Simple + Complex Reaction Time	Processing speed

Table 1 is broken up into groups featuring either a global cognitive function test and a premorbid intellectual function test, or domain-specific cognitive tests. Test names are listed on the right, and the test domain for each test is listed in the right column. These test include montral cognitive assessment (MoCA) (Rossetti et al. 2011), North American adult reading test (NAART) (Fromm et al. 1991; Bolla et al. 1998), mnemonic similarity test (MST) (Stark et al. 2013; 2019), Face-Name associative memory exam (FNAME) (Amariglio et al. 2012), Verbal parried associative Learning (VPA) (Clark et al. 2018), Number letter (Rogers and Monsell 1995; Glisky et al. 2020), flanker (Eriksen and Eriksen 1974), keep Track (Glisky et al. 2020), rey auditory Verbal Learning test (AVLT) (Moradi et al. 2017), deary simple + complex reaction time (deary) (Deary et al. 2011), and blobs (Ryan et al. 2012).

**Table 2.** Biomarker stratification table.

	Groupings		
Biomarker	Age	Measured values	Assigned Values
**NfL**	≤50	≤7 pg/mL	1
		>7-<11 pg/mL	2
		≥11 pg/mL	4
	51–74	≤10.5 pg/mL	1
		>10.5-<17.75 pg/mL	2
		≥17.75 pg/mL	4
	≥ 75	≤16 pg/mL	1
		>16-<26 pg/mL	2
		≥26 pg/mL	4
**IL-6**	All Ages	>1.18 pg/mL	1
		1.18–1.74 pg/mL	2
		>1.75 pg/mL	4
**TNFα**	All Ages	<80 pg/mL	1
		80–120pg/mL	2
		>120pg/mL	4
**NT-proBNP**	All Ages	<125 pg/mL	1
		125–300 pg/mL	2
		>300 pg/mL	4

Table 2 illustrates the assigned calculated scores for each component and age group for plasma measured biomarkers, including neurofilament light protein (NfL) interleukin-6 (IL-6), tumor necrosis factor-alpha (TNFα), N-terminal pro-B-type natriuretic peptide (NT-proBNP).

**Table 3.** Background of test groups stratified by NT-proBNP levels.

	Control	HF NT-proBNP 0–499 pg/mL	HF NT-proBNP >500 pg/mL	*p* value Anova or T-Test
**Demographics**				
**Age**	64.41 ± 8.36 n = 34	67.71 ± 10.35 n = 14	67.64 ± 9.52 n = 11	0.3982
**Sex (Male%)**	45.50%	57.10%	81.80%	#0.0231*
**Race and Ethnicity**				
Non-Hispanic Caucasian	84.40%	64.30%	90.90%	#0.3398
Hispanic	12.50%	28.60%	9.10%	#0.4946
Non-Hispanic African American	3.10%	0.00%	0.00%	#0.1137
Asian	0.00%	0.00%	0.00%	>0.9999
Other	0.00%	7.10%	0.00%	#0.4545
**Education**	16.42 ± 1.68 n = 12	15.93 ± 2.13 n = 14	13.91± 2.17 n = 11	0.0139*
**Comorbidities**				
**Diabetic**	NA #	15.38%	66.67%	#0.0260*
**CKD**	NA #	16.67%	62.50%	#0.0623
Creatinine	NA #	0.99 ± 0.19 *n* = 11	1.26 ± 0.39 *n* = 5	0.0757
BUN	NA #	18.27 ± 5.04 *n* = 11	18.00 ± 9.90 *n* = 2	0.9511
eGFR	NA #	73.09 ± 15.76 *n* = 11	61.80 ± 25.41 *n* = 5	0.2897
**CAD**	NA #	61.50%	37.50%	#0.3870
**Atrial fibrillation/Flutter**	NA #	46.20%	57.10%	#>0.9999
**COPD/lung disease**	NA #	23.10%	0.00%	0.1329
**Malignancy**	NA #	15.40%	22.90%	#>0.9999
**Systemic HTN**	NA #	50.00%	66.70%	#0.6605
**Heart-Based Measurements**				
**Average EF%**	NA #	40.25 ± 13.27 *n* = 14	24.78 ± 8.423 *n* = 9	0.0054**
**NYHA Classification Range**	NA #	II-III	II-IV	0.4433
**Medications**				
**Beta-blocker**	NA #	92.30%	88.80%	#>0.9999
**RAASi**	NA #	92.30%	77.78%	#0.5442
**MRA**	NA #	69.20%	87.50%	#0.6065
**SGLT2i**	NA #	46.15%	77.77%	#0.2031
**Loop diuretic**	NA #	23.08%	75.00%	0.0183*

Table 3 lists the demographics, comorbidities, heart-based measurements, and medications taken by our three test groups. Table 3 is separated into three groups: one control, and two heart failure (HF) groups. Individuals with HF are stratified into two groups by N-terminal pro-B-type natriuretic peptide (NT-proBNP) levels, ranging from 0 pg/mL to 499 pg/mL, and ≥500pg/mL. NA control individuals are of healthy control values as assumed to be within a normal healthy range. Values that are represented in ratios are presented as percentages of individuals within the corresponding NT-proBNP group. Other values are represented as average ±standard deviation, n = number of individuals. Groups were tested for normality via d’Agostino & pearson test or Shapiro-Wilk test if n was too low. Values that compare three groups and meet normality were tested for significance via ANOVA, and values that do not meet normality were tested for significance via Kruskal-Wallis test. Values that compare two groups and meet normality were tested for significance via student t test, and values that do not meet normality were tested for significance via Mann-Whitney test indicated with at # preceding the p-value. Chronic kidney disease (CKD), blood urea nitrogen (BUN), estimated glomerular filtration rate (eGFR), coronary artery disease (CAD), chronic obstructive pulmonary disease (COPD), hypertension (HTN), ejection fraction (EF), New York heart Association (NYHA), renin-angiotensin-aldosterone system inhibitors (RAASi), mineralocorticoid receptor antagonist (MRA), sodium-glucose cotransporter-2 inhibitors (SGLT2i). P value indicated by *.

* *p*=0.05–0.01

** *p* = 0.009–0.001

*** *p*=0.0009–0.0001

**** *p* < 0.0001.

**Table 4.** HF participants perform worse on cognitive assessment.

Test		Control Mean ± SDV, *n*=	HF Mean ± SDV, *n*=	*p* value T-Test
Cognitive and Premorbid Function Tests				
MoCA				
	MoCA Total	27.800 ± 1.687, 10	24.290 ± 3.008, 33	0.0011**
	MoCA Age/education z-score	0.926 ± 0.725, 10	−0.439 ± 1.376, 32	0.0047**
NAART				
	NAART Total Correct	49.000 ± 5.812, 10	37.788 ± 8.799, 33	#0.0001***
	NAART Age z-score	0.315 ± 0.942, 10	1.841 ± 1.224, 32	#0.0008***
	Verbal IQ	118.020 ± 5.173, 10	108.176 ± 8.059, 33	#0.0002***
	Full-Scale IQ	119.653 ± 2.561, 9	109.772 ± 7.153, 32	0.0002***
Domain-specific Cognitive assessments				
FACE NAME				
	Cued Name Retrieval (CRN)	10.500 ± 1.841, 10	6.531 ± 2.828, 32	#<0.0001****
	Cued Occupation Retrieval (CRO)	11.500 ± 0.707, 10	10.355 ± 1.704, 31	0.0467*
	Facial Recognition	12.000 ± 0.000, 11	11.939 ± 0.242, 33	0.4151
	Cued Name Retrieval 30-minute Delay (CRN30)	10.400 ± 1.776, 10	6.656 ± 2.847, 32	#0.0002***
	Cued Occupation Retrieval 30-minute Delay (CRO30)	11.600 ± 0.699, 10	10.313 ± 1.731, 32	#0.0328*
	Multiple Choice Names (MCN)	11.778 ± 0.441, 9	10.161 ± 1.573, 31	0.0045**
	Multiple Choice Occupations (MCO)	12.000 ± 0.000, 10	11.750 ± 0.440, 32	#0.1646
	FNN	38.300 ± 6.897, 10	22.939 ± 9.880, 33	#<0.0001****
	FNO	44.200 ± 3.120, 10	38.750 ± 6.217, 32	#0.0037**
	Total FNAME Score	81.800 ± 11.233, 10	62.344 ± 13.804, 32	#<0.0001****
Keep Track				
	Keep Track Total	23.600 ± 3.658, 10	17.688 ± 3.914, 32	0.0001***
VPA				
	VPA: Total	26.111+ 4.428, 9	17.364 ± 5.851, 33	0.0002***
Actual Composite Cognitive Score				
Actual Composite				
Cognitive Score	Actual Composite Cognitive Score: CRN+CRN30 + CRO+CRO30+VPA+Keep Track	0.776 ± 4.099, 10	−5.480 ± 4.841, 32	#0.0001***
Background				
	Age	63.818 ± 8.134, 11	68.618 ± 8.931, 34	0.1212
	Sex	64% Male	38% Male	#0.0165*
	Education	16.455 ± 1.753, 11	15.294 ± 2.250, 34	#0.1332

Cognitive and premorbid functional assessments listed include the montreal cognitive assessment (MoCA) and the North American adult reading test (NAART). Values for each test, including total raw score and age score, are listed under each assessment. Cognitive assessments listed include face name (FNAME), keep track, and verbal Paired associates (VPA); the tasks associated with each cognitive assessment are listed under the corresponding assessment. A composite score was calculated (Actual Composite cognitive Score) from z-score transformed cognitive assessment variables to identify impairment in cognitive performance related to FNAME, keep Track, and VPA. Age, sex, and education for all people tested in the neurophysiological test are presented last in the table. Values for heart failure (HF) participants and control participants are presented as average ± standard deviation, n = number of individuals. Groups were tested for normality via d’Agostino & pearson test or Shapiro-Wilk test if n was too low. Values that meet normality were tested for significance via the student t-test, and values that did not meet normality were tested for significance via the Mann-Whitney test indicated with at # preceding the p value. P value indicated by *.

* *p* = 0.05–0.01

** *p* = 0.009–0.001

*** *p*=0.0009–0.0001

**** *p* < 0.0001.

**Table 5.** Significant blood biomarkers.

	Control	HF	
	Mean ± SDV, *n*=	Mean ± SDV, *n*=	*p* value
Neurodegenerative Biomarkers			
NfL	12.9511 pg/mL ± 5.86607, 19	18.8395 pg/mL ± 8.75971, 21	0.0181*
pTau181	1.093 pg/mL ± 0.703, 13	1.320 pg/mL ± 0.952, 20	# 0.6961
pTau217	NA	0.281 pg/mL ± 0.176, 28	NA
PlGF	8.201 pg/mL ± 1.788, 23	8.810 pg/mL ± 1.386, 24	0.1976
Cytokines			
IL-6	0.9677 pg/mL ± 0.5429, 24	2.562 pg/mL ± 2.068, 25	# <0.0001****
IL-12p40	49.4532 pg/mL ± 23.0791, 25	122.523 pg/mL ± 79.8448, 26	<0.0001****
IL-15	5.731 pg/mL ± 2.396, 25	9.639 pg/mL ± 6.140, 25	0.0047**
MIP-1α	7.9334 pg/mL ± 7.2211, 25	22.364 pg/mL ± 22.795, 26	#0.0067**
TNFα	50.561 pg/mL ± 18.99818, 25	82.57266 pg/mL ± 35.13265, 26	0.0002***
TNFβ	23.723 pg/mL ± 11.269, 25	34.318 pg/mL ± 22.660, 26	#0.0259*
Composite Biomarker Cognitive Score			
Composite Biomarker Cognitive Score	5.09091 ± 0.83121, 11	10.0000 ± 3.08957, 23	<0.0001****
NFL+NT-proBNP + IL-6+TNFα			

Values of serum biomarkers that are higher in heart failure (HF) participants compared to control participants are listed in the table. Neurodegenerative serum biomarkers include neurofilament light protein (NfL), phosphorylated tau 181 (pTau181), phosphorylated tau 217 (pTau217), and placental growth factor (PlGF). Cytokines listed in the table include interleukin (IL)-6, IL-12p40, IL-15, macrophage inflammatory protein-1 alpha (MIP-1α) (CCL3), tumor necrosis factor-alpha (TNFα), and tumor necrosis factor-beta (TNFβ). Stratified blood biomarkers are added to create the Composite Biomarker Cognitive Score. The values for heart failure (HF) participants and control participants are presented as average ±standard deviation, n = number of individuals. Groups were tested for normality via d’Agostino & pearson test or Shapiro-Wilk test if n was too low. Values that meet normality were tested for significance via the student’s t-test, and values that did not meet normality were tested for significance via the Mann-Whitney test indicated with at # preceding the *p* value.

* *p* = 0.05 to 0.01

** *p* = 0.009–0.001

*** *p* = 0.0009–0.0001

**** *p* < 0.0001.

**Table 6.** Correlation table of the Composite Biomarker Cognitive Score and cognitive assessments.

Calculated Biomarker Cognitive Score		
NFL+NT-proBNP + IL-6+TNFα		
	Pearson r	*p* value
Actual Composite Cognitive Score	−0.5948	0.0002***
Cognitive Assessments		
CRN	−0.4926	0.0031**
CRO	−0.497	0.0028**
CRN30	−0.5776	0.0003***
CRO30	−0.4365	0.0099**
MCN	−0.3779	0.0275*
MCO	−0.4124	0.0154*
FNN	−0.5259	0.0014**
FNO	−0.4738	0.0046**
FNAME	−0.5566	0.0006***
VPA Total	−0.4131	0.0169*
Keep Track	−0.3996	0.0192*
Cognitive and Premorbid Function Test		
MoCA total score	−0.3854	0.0244*
MoCA Age/Education z-score	−0.2821	0.106
NAART Total	−0.3904	0.0224*
NAART Total Age z-score	0.265	0.1298
Verbal IQ	−0.3644	0.0341*

Pearson r values and significance are listed for interaction for the Composite Biomarker cognitive Score vs. The actual Composite cognitive Score, cognitive assessment tasks, and functional assessment scores. Correlation values are also listed for the composite score vs cognitive assessment tasks, and premorbid functional assessment scores.

* *p* = 0.05–0.01

** *p* = 0.009–0.001

*** *p* = 0.0009–0.0001

**** *p* < 0.0001.

**Table 7.** Preliminary results show a decrease in cognitive abilities.

	HF Baseline	HF Follow-up
Test	(Mean ± SDV, *n*=)	(Mean ± SDV, *n*=)
Moca		
Moca age/education z-score	0.579 ± 0.586, 5	−0.300 ± 1.026, 5
MST		
Lure Discrimination Index	0.172 ± 0.205, 5	0.034 ± 0.038, 5
AVLT		
Short Delay Free Recall List A (MOAN SS)	1.001 ± 1.613, 6	−0.113 ± 1.528, 6
Long Delay Free Recall List A (MOAN SS)	0.815 ± 1.655, 6	−0.013 ± 1.140, 6

5 heart failure (HF) participants were followed longitudinally for 12 months. The cognitive tests listed were trending or significantly different from the control during follow-up but not at baseline. Values listed at baseline are from the same individuals as values listed at 12-month follow-up. Values are represented as average ± standard deviation, n: number of individuals.

**Table 8.** Preliminary results show an increase in serum cytokine levels in HF participants

	HF Baseline	HF Follow-up	
Cytokine	(Mean ± SDV, *n*=)	(Mean ± SDV, *n*=)	*p* value
Eotaxin	121.414 ± 35.477, 5	131.975 ± 38.873, 5	0.0709
IFNy	117.428 ± 52.052, 5	172.221 ± 132.240, 5	#0.0625
IL-1a	34.244 ± 48.150, 5	66.643 ± 98.906, 5	#0.1250
IL-1b	41.640 ± 42.658, 5	96.836 ± 109.115, 5	#0.1981
IL-4	4.774 ± 4.380, 5	10.225 ± 8.623, 5	0.172
IL-6	4.430 ± 4.519, 5	7.854 ± 8.024, 5	0.1179
IL-12p40	171.406 ± 120.900, 5	1499.9 ± 2899.70, 5	#0.1250
IL-17	26.264 ± 44.137, 5	72.265 ± 116.593, 5	#0.1250
MIP-1a	35.896 ± 30.809, 5	56.029 ± 44.666, 5	0.1592
TNFα	45.572 ± 15.277, 5	70.147 ± 53.656, 5	#0.1875
TNFβ	42.554 ± 31.087, 5	126.539 ± 172.881, 5	#0.0625

5 heart failure (HF) participants were followed longitudinally for 12 months. The cytokines listed were trending or significantly different from the control during follow-up but not at baseline. Values listed at baseline are from the same individuals as values listed at 12-month follow-up. Values are represented as average ± standard deviation, n: number of individuals. Groups were tested for normality via d’Agostino & Pearson test or Shapiro-Wilk test if n was too low. Values that meet normality were tested for significance via the student’s t-test, and values that did not meet normality were tested for significance via the Wilcoxon test, indicated with a # preceding the *p* value. *p* = 0.05–0.01*.

## Data Availability

The data that support the findings of this study are available upon reasonable request from the corresponding author.

## CLINICAL SIGNIFICANCE

While previous reports have shown that patients with HF are highly likely to have MCI and increased levels of neurodegeneration biomarkers such of NfL and pTau181, in this study, for the first time, we have created a Composite Biomarker Cognitive Score, encompassing neurodegenerative serum biomarkers, NfL, proinflammatory serum cytokine, IL-6, and TNFα, and HF biomarker NT-proBNP to predict an individual’s cognitive performance. Our results indicate that cognitive impairment in HF may be predicted using a combination of neurodegeneration, inflammatory, and cardiac function biomarkers and may be useful in stratifying patients who may benefit from cognitive protective therapies.
