# Supplementary material for: A novel composite biomarker score for the identification of cognitive impairment in patients with heart failure: a pilot study
Source: Biomarkers. Author manuscript; Available in PMC 2026 Jan 26. (PMC12834611; doi:10.1080/1354750X.2025.2585003)
Supplement: Supp 1 [file NIHMS2126198-supplement-Supp_1.pdf]

# Supplemental Methods:

## Montreal Cognitive Assessment (MoCA):

The Montreal Cognitive Assessment is a test that measures global cognitive function (Rossetti et al. 2011). It is used to detect mild cognitive impairment and general cognitive function. It tests attention, verbal memory, language, orientation, visual spatial, and executive function (Rossetti et al. 2011). Attention is assessed via the target detection task, the serial subtraction task, and repeating numbers forward and backward. Language abilities are determined by a task where the individual is asked to name three common animals from an image, and repetition of sentences. To measure verbal fluency, an individual is asked to name as many words as possible starting with the letter "F" within 60 seconds. Orientation is tested by asking the participant the time, date, and place they are located (Nasreddine et al. 2005). Visual spatial abilities are determined based on the participant's ability to draw a clock at a certain time, and a 3D cube. Executive functions are assessed via a trail-making test, a two-item verbal abstract task, and a verbal fluency task. Individuals' performance on each test is scored and added together for the total MoCA score. Total scores are normalized to age and education and represented as the MoCA z-score using Rossetti et al., 2011 for participants under 70 (Rossetti et al. 2011) and Malek-Ahmadi et al., 2015 for participants over 70 (Malek-Ahmadi and Nikkhahmanesh 2024).

## North American Adult Reading Test (NAART):

Individuals are prompted to read a list of 60 words that are commonly pronounced irregularly (Bolla et al. 1998). Scores are based on how many words an individual reads/pronounces correctly. This tests semantic fluency, which has been shown to be impaired in dementia and correlated with the severity of dementia in Alzheimer's disease (Fromm et al. 1991). Previous studies have shown the NAART to estimate premorbid mental abilities of individuals with mild to moderate dementia (Fromm et al. 1991). The NAART relies heavily on

Information Classification: General

an individual's reading decoding skills and crystallized abilities. The participant is presented with a sheet of 61 words in four columns. Each column increases in difficulty. The participant is asked to read the word out loud correctly. The total correct score is z-scored transformed for age and gender using Bolla et al., 1998 (Bolla et al. 1998). The Verbal IQ and Full-scale IQ were calculated using the number of words the participants incorrectly pronounced.

**Verbal IQ= 128.7-0.89*(number incorrectly pronounced).** **Equation 1**

**Full-Scale IQ=127.8-0.78*(number incorrectly pronounced).** **Equation 2**

## Face Name (FNAME):

The FNAME is an associative memory assessment that tests an individual's abilities in learning, recognition, recall, and matching (Fox et al. 2022); it measures how many faces are accurately recognized and names and professions recalled (Amariglio et al. 2012). FNAME was presented to participants on the computer using Microsoft PowerPoint. Faces were presented to the participants one at a time. Each face was presented for 8 seconds. During the learning phase, participants were asked to read out loud and to remember the names and occupations associated with the corresponding face on the screen. Faces seen during the learning phase were then presented to the participant, and the participant was asked to recall the name and occupation that corresponded to the face presented on the screen (trial 1). Following the recall phase of trial 1, participants were again asked to read out loud and to remember the names and occupations associated with the corresponding face on the screen. Faces in the second learning phase are the same but presented in a different order. Participants are then asked to recall the names and occupations of the faces presented during the recall phase in trial 2. The number of names correctly recalled for Trial 1 is the Initial Name Recall (INR1), and Trial 2 is the Initial Name Recall (INR2). The number of occupations correctly recalled for Trial 1 is the Initial Occupation Racal (IOR1), and Trial 2 is the Initial Occupation Racal (IOR2). After a ~5-minute delay (short delay), individuals are asked to recall the names (CRN) and occupations (CRO) of

Information Classification: General

the previous faces they had learned in trials 1 and 2. After 30 minutes, participants are presented with three faces. One of the three faces they have seen during the learning phase, the other two faces share similar age, ethnicity, and sex. Participants are asked to indicate the face they had previously seen in the learning phases (facial recognition). The correct face is then presented to the participant, and they are then asked to recall the name (CRN30) and occupation (CRO30) of the individual. Following their response, the same face is presented with three names (Multiple Choice Names- MCN) and occupations (Multiple Choice Occupation-MCO) for the participant to choose from. The total correct names and occupations recalled correctly over all trials were added to calculate the FNN (**Equation 1**) and FNO (**Equation 2**). The total score of correct name and occupation recall for the learning trials, short delay, and long delay, is used as the FNAME score (**Equation 3**).

**FNN= INR1+INR2+CRN+CRN30** **Equation 1**

**FNO= INO1+INO2+CRO+CRO30** **Equation 2**

**FNAME=FNN+FNO** **Equation 3**

### Verbal Paired Associates (VPA):

The VPA task is used to assess verbal memory (Clark et al. 2018; Talboom et al. 2019). Previous studies have shown that hippocampal damage results in impaired performance in the VPA task (Clark et al. 2018). Scores for the VPA are expressed as a percentage of words that were correct over three trials. VPA is presented to the participant on the computer using E-Prime software. During this task, an individual is given a list of 12-word pairs. During the learning phase 12-word pairs appear on the screen for 2 seconds each. After, participants are asked to recall the associated word that pairs with a prompted word they had previously seen during the learning phases. These word pairs are presented again in a different order for two more trials with a recall phase proceeding immediately after the learning phase. The primary dependent measure is the total words recalled across all three trials (total of 36).

Information Classification: General

**Rey Auditory Verbal Learning Test (AVLT):**

The AVLT is useful in assessing episodic memory and is sensitive to verbal memory deficiencies (Moradi et al. 2017). Participants were read a list of words at an approximate pace of one word per second. Lists were read out loud and were then immediately repeated back to the administrator after the list had been read. List A consisted of 15 words and was read and repeated back in a total of 5 consecutive trials (List A Trials 1-5). List B is then read to the participant and asked to be repeated back immediately. List B consists of 15 new words. Following List B, the participant is asked to recall words from the first list (List A, Trial 6). After 30 minutes, participants are asked to recall the words from the first list (List A, Trial7). After recall, participants are asked to circle all the words that were on List A on a sheet listing 50 words that contain words from List A. Lists used were obtained from Geffen et al. 1994 (Geffen et al. 1994). An alternate list was given to the participants for follow-up visits.

"List A Total" reflects how many total words the participant can recall after during the 5 consecutive trials of List A (Trial 1-5). It is scored by summing the total number of correct responses for List A Trial 1-5. "Learning over time" (LOT) measures learning over the 5 consecutive trials from List A. LOT= (Trial1+Trial2+Trial3+Trial4+Trial5) – (5x Trial1). "List A Short Del Free Rec" is the total number that participant recalled correctly from List A on Trial 6. The number correctly recalled after 30 minutes makes up the score for "List A Long Del Free Rec". "Recognition Hits" is the total number of correct words circled on the sheet containing 50 words. Values were normalized using the Geffen (1994) normed values according to sex and age indicated by (MOANS SS) (Geffen et al. 1994).

**Mnemonic Similarity Task (MST):**

The Mnemonic Similarity Task (MST) assesses object recognition and pattern separation domains and are well known to rely on the hippocampus (Stark et al. 2013). Detailed description and methodology can be found in the paper by Shauna et al. (Stark et al. 2019; Stark et al.

2013); however, a brief methodology is explained here in the supplementary methods. There are two phases of the MST. In the first phase (study phase), participants are presented with 128 pictures of objects on a white background, one at a time, and are tasked to determine whether the image is commonly found indoors or outdoors. During this phase, the participant engages in an incidental encoding task (Stark et al. 2019). During the second phase (test phase), the participants are presented with objects again but are tasked to identify if the object is “Old”, “Similar”, or “New” compared to objects presented in the first phase. Both phases present objects for two seconds, followed by an inter-trial interval of 0.5 seconds. The images presented in the test phase are either the exact repetitions of image presented in the study phase (indicated as “targets” in results); a new image not previously seen (indicated as “foils” in results); or are perceptually similar but not identical to images seen during the study phase (indicated as “lures” in results). One third of the images in the test phase are target images, one third of the images are foils images, and the final third of the images are lures. The total correct responses are the total percent corrected for omissions. Correct recognition was calculated by **Equation 1**, and the Lure discrimination ratio was calculated by **Equation 2**.

**Correct Recognition=(“Old-Target Percent Endorsed”-“Old-Foil Percent Endorsed”)/100**

**Equation 1**

**Lure Discrimination Ratio=(“Similar-Lure Percent Endorsed”-“Similar-Foil Percent Endorsed”)/100**

**Equation 2**

## Blobs:

A detailed description of Blobs is described in a publication by Ryan et al. (Ryan et al. 2012). This task was completed on the computer using E-Prime. Participants are shown two stimuli simultaneously on the screen and must decide if they are a match or a non-match. Participants press “1” key on the keyboard if it is a match or press “2” if it is a non-match.

Information Classification: General

Participants are told that the stimuli will always be rotated and to focus on features of the stimuli. Stimuli included blobs or squares. Blobs were characterized by three features: an inner shape, an outer shape, and the fill in between. Blobs are considered a non-match if they differ on any one of those features. As a perceptual control task, participants were also shown squares that could be identical in size (match) or vary in size (non-match). Discrimination of the stimuli varied on the level of difficulty. Easy, non-matching blob pairs differed in all three features, while easy squares differed by a size of 5-8 mm. Hard, non-matching blob pairs differed in only one feature (inner shape, outer shape, or fill) while hard squares differed by a size of 2-4 mm. A practice session of 21 self-paced trials with feedback. The actual task consisted of 200 trials: 25 hard blobs, 25 hard squares, 25 easy blobs, 25 easy squares, and 100 matching pairs. Trials automatically moved on after 5 seconds. Primary dependent measures include proportion correct for easy and hard blobs and squares.

### Flanker:

Flanker was administered on the computer using Psytoolkit Flanker Inhibitory Control and Attention Test (https://www.psytoolkit.org/experiment-library/flanker.html). The flanker test measures inhibitory control and attention (Eriksen and Eriksen 1974). The participant is directed to use the keyboard to indicate the direction of the middle stimuli on the screen. The arrow is surrounded by other arrows that are either going the same direction (congruent trial), or the flanking arrows are pointing in the opposite direction of the middle arrow (incongruent trial). Congruent and incongruent are presented in a quasi-random order. The participant is asked to first participate in a practice round, which is followed by the test. Participants have 5 seconds per trial. No answer during the trial is marked as incorrect, and then progresses automatically to the next trial. The Flanker effect was calculated as the reaction time (ms) for correct responses on the incompatible trials subtracted by compatible trials. Percent accuracy is the number that the participant got correct.

### Number Letter:

The Number-Letter task was adapted from Roget et al. (Rogers 1995). Detailed description and methodology can be found in the paper by Glinsky et al. (Glisky et al. 2020); however, a brief methodology is explained here in the supplementary methods. During this task, the participant is presented with a number-letter pair (e.g., 8G) on a computer using E-Prime software. Participants were asked to determine if the number was odd or even, or if the letter was a vowel or a consonant. The number-letter pairs are presented on the computer screen in one of four quadrants. The location of the number-letter pairs appears determines whether the participant responds to the number or letter. If the stimulus is in the top two quadrants, participants were to distinguish if the number was odd or even by pressing the “1 or “2” key with the right hand on the keyboards number pad. If the stimulus was in the bottom two quadrants, the participants were to distinguish if the letter was a consonant or vowel by pressing the “c” or “v” key with the left hand on the keyboard. There are three blocks: 1) number-letter pairs only appear on half of the screen (32 trials), 2) number-letter pairs only occur on the bottom half of the screen (32 trials), 3) number-letter pairs appear in all quadrants, rotating in a clockwise order, starting at the top left-hand corner (64 trials). Before performing each block, the participant participated in a practice trial for each discrimination task. The dependent measure was the global-shift cost: the difference between the average RTs for the shift block and the average RTs across the two no-shift blocks. To control for differences in baseline speed of responding, we used a proportional measure, dividing the difference scores by the average RTs for the no-shift blocks.

### Keep Track:

Keep track is presented to participants on the computer using E-Prime software. Keep Track evaluated updating and working memory (Glisky et al. 2020). Detailed description and methodology can be found in the paper by Glinsky et al. (Glisky et al. 2020); however, a brief methodology is explained here in the supplementary methods. In this task, participants are presented with a list of words and are tasked with remembering the last-presented word from

the assigned categories. The participants are first familiarized with the six categories (distances, fruits, furniture, metals, relatives, sports) and the words associated with each category. Participants are then shown a list of 15 words from multiple categories, one word at a time in the center of the computer screen. Words are shown for 1500 ms. They are instructed to remember the last word in a specific category. The number of categories that they are instructed to keep track of increases with each trial, starting with one category and up to 4. With each category, the participant completes three trials. Target category names remain at the bottom of the screen throughout each trial for reference. Performance was determined based on the number of correct responses across all trials (out of a possible 30).

### Deary Simple + Complex Reaction Time (Deary):

The Deary tests processing speed (Deary et al. 2011). A detailed description of the Deary is described in a publication by Deary et al. (Deary et al. 2011). In brief, the Deary is given to participants on the computer. A practice section precedes each tested task. In the first task, an “X” appears in the middle of the screen for the simple reaction time task. The participant is asked to press the space bar with their dominant hand when the “X” appears on the screen. Premature hits were considered incorrect. In the second task, an “X” appears in one of the 4 boxes for the choice reaction time task. When the “X” appears in one of the boxes, the participant is to press the correct corresponding key to indicate the box the “X” has appeared. The Simple Reaction Time Percent Correct was calculated as a percentage of occurrences that were considered correct (times the participants responded after stimuli were presented). The Simple Reaction Time Mean Reaction Time (RT) is the average reaction time for a participant to respond to stimuli. The Choice Reaction Time Percent Correct was calculated as a percentage of occurrences that were considered correct for the choice reaction time tasks. The Choice Reaction Time Mean RT is the average reaction time for a participant to respond to stimuli during the choice reaction time tasks.

## Supplemental Table and Legend:

## Supplemental Table 1

| | | Control Mean ± SDV, n= | HF Mean ± SDV, n= | T Test p-value |
|---|---|---|---|---|
| **Cognitive and Premorbid Function Tests** | | | | |
| **MoCA** | | | | |
| | **MoCA Total** | 27.800±1.687, n=10 | 24.288±3.008, n=33 | 0.0011** |
| | **MoCA Age/ education z-score** | 0.926±0.725, n=10 | -0.439±1.376, n=32 | 0.0047** |
| **NAART** | | | | |
| | **NAART Total Correct** | 49.000±5.812, n=10 | 37.250±8.371, n=32 | #<0.0001**** |
| | **NAART Age z-score** | 0.315±0.942, n=10 | 1.841±1.224, n=32 | #0.0008*** |
| | **Verbal IQ** | 118.020±5.173, n=10 | 108.176±8.059, n=33 | #0.0002*** |
| **Domain-Specific Cognitive Assessments** | | | | |
| **FACE NAME** | | | | |
| | **Cued Name Retrieval (CRN)** | 10.500±1.841, n=10 | 6.531±2.828, n=32 | #<0.0001**** |
| | **Cued Occupation Retrieval (CRO)** | 11.500±0.707, n=10 | 10.355±1.704, n=31 | 0.0467* |
| | **Facial Recognition** | 12.000±0.000, n=11 | 11.939±0.242, n=33 | 0.4151 |
| | **Cued Name Retrieval (CRN30)** | 10.400±1.776, n=10 | 6.656±2.847, n=32 | #0.0002*** |
| | **Cued Occupation Retrieval (CRO30)** | 11.600±0.699, n=10 | 10.313±1.731, n=32 | #0.0328* |
| | **Multiple Choice Names (MCN)** | 11.778±0.441, n=9 | 10.161±1.573, n=31 | 0.0045** |
| | **Multiple Choice Occupations (MCO)** | 12.000±0.000, n=10 | 11.750±0.440, n=32 | #0.1646 |
| | **FNN** | 38.300±6.897, n=10 | 22.939±9.880, n=33 | #<0.0001**** |
| | **FNO** | 44.200±3.120, n=10 | 38.750±6.217, n=32 | #0.0037** |
| | **Total FNAME Score** | 81.800±11.233, n=10 | 62.344±13.804, n=32 | #<0.0001**** |
| **VPA** | | | | |
| | **VPA: Total** | 26.111±4.428, n=9 | 17.364±5.851, n=33 | 0.0002*** |
| **Keep Track** | | | | |
| | **Keep Track Total** | 23.600±3.658, n=10 | 17.688±3.914,n= 32 | 0.0001*** |
| **AVLT** | | | | |
| | **List A Total (MOANS SS)** | -0.0482±1.713,n=11 | -0.161±1.207,n=32 | 0.8123 |
| | **Learning Over Time (MOANS SS)** | 10.727±3.165,n=11 | 9.969±2.741,n=32 | 0.4508 |
| | **List A Short Del Free Rec (MOANS SS)** | 0.163±1.396,n=11 | -0.272±1.292 ,n=32 | 0.3515 |
| | **List A Long Del Free Rec (MOANS SS)** | -0.086±1.214,n=11 | -0.407±1.304,n=32 | 0.4784 |
| | **Recognition Hits z-score** | 0.253±0.848,n=11 | -0.162±1.044,n=31 | 0.2434 |
| **MST** | | | | |
| | **Correct Recognition Memory** | 0.873±0.073, n=11 | 0.825±0.096, n=30 | 0.1463 |
| | **Percent correct** | 68.877±4.540, n=10 | 65.122±8.527, n=32 | 0.192 |
| | **Lure Discrimination Index** | 0.239±0.131, n=11 | 0.159±0.187, n=31 | 0.1993 |
| | **Old-Target Percent Endorsed** | 89.000±6.340, n=11 | 84.781±10.450, n=32 | #0.2963 |
| | **Old-Lure Percent Endorsed** | 58.000±11.498, n=11 | 57.094±14.632, n=32 | 0.8533 |
| | **Old-Foil Percent Endorsed** | 1.800±1.317, n=10 | 3.774±2.963, n=31 | 0.0493* |
| **Number-Letter** | | | | |
| | **Overall Global Shift** | 370.957±84.230, n=10 | 556.227±254.393, n=31 | 0.0304* |
| | **Overall Local Shift** | 475.524±133.516, n=11 | 554.099±321.838,n=32 | 0.4388 |
| **Flanker** | | | | |
| | **Flanker Effect (ms)** | 90.844±35.696, n=11 | 85.939±72.278, n=33 | #0.9096 |
| | **Accuracy** | 99.200±1.033, n=10 | 97.517±2.487, n=29 | #0.0393* |
| **Deary Simple + Complex Reaction Time** | | | | |
| | **Simple Reaction Time Percent Correct** | 1.000±0.000, n=8 | 1.000±0.000 n=32 | #>0.9999 |
| | **Simple Reaction Time Mean RT** | 346.030±35.476, n=10 | 368.692±70.183, n=30 | 0.336 |
| | **Choice Reaction Time Percent Correct** | 99.278±1.093, n=9 | 99.183±1.178, n=30 | #0.7199 |
| | **Choice Reaction Time Correct Mean RT** | 598.257±52.470, n=9 | 688.027±105.800, n=31 | 0.0193* |
| **Blobs** | | | | |
| | **Overall Percent Accuracy** | 78.778±4.549, n=9 | 72.879±6.766, n=29 | 0.0198* |
| | **Hard Blobs Percent Accuracy** | 63.700±10.833, n=10 | 56.359±7.727, n=29 | 0.0253* |
| | **Easy Blobs Percent Accuracy** | 82.333±5.657, n=9 | 78.695±8.134, n=30 | 0.2196 |
| | **Hard Size Percent Accuracy** | 71.333±7.810, n=9 | 68.389±10.113, n=30 | 0.4277 |
| | **Easy Size Percent Accuracy** | 92.556±6.617, n=9 | 92.146±7.699, n=28 | 0.8681 |
| | **Average Reaction Time** | 2285.880±205.764, n=9 | 2286.509±338.043, n=29 | 0.9958 |

**Supplemental Table 1: List of all cognitive assessments.** Cognitive and premorbid functional assessments listed include the Montreal Cognitive Assessment (MoCA) and the North American

Adult Reading Test (NAART). Values for each test, including total raw score and z scores score, are listed under each functional assessment. Cognitive assessments listed include Face Name (FNAME), Verbal Paired Associates (VPA), Keep Track, Rey Auditory Verbal Learning Test (AVLT), Mnemonic Similarity Task (MST), Number-Letter, Flanker, Deary Simple + Complex Reaction Time, and Blobs; the tasks associated with each cognitive assessment are listed under the corresponding assessment. Values for heart failure (HF) participants and control participants are presented as average ±stand deviation, n=number of individuals. Groups were tested for normality via D'Agostino & Pearson test or Shapiro-Wilk test if n was too low. Values that meet normality were tested for significance via the student t-test, and values that did not meet normality were tested for significance via the Mann-Whitney test indicated with at # preceding the p value. Corresponding age, sex, and education of all people tested in the neurophysiological test are presented at the bottom of Table 4.

Information Classification: General

# Reference:


Amariglio RE, Frishe K, Olson LE, Wadsworth LP, Lorius N, Sperling RA, Rentz DM. 2012. Validation of the face name associative memory exam in cognitively normal older individuals. Journal of Clinical and Experimental Neuropsychology. 34(6):580-587.

Bolla KI, Gray S, Resnick SM, Galante R, Kawas C. 1998. Category and letter fluency in highly educated older adults. The Clinical Neuropsychologist. 12(3):330-338.

Clark IA, Kim M, Maguire EA. 2018. Verbal paired associates and the hippocampus: The role of scenes. Journal of Cognitive Neuroscience. 30(12):1821-1845.

Deary IJ, Liewald D, Nissan J. 2011. A free, easy-to-use, computer-based simple and four-choice reaction time programme: The deary-liewald reaction time task. Behavior Research Methods. 43(1):258-268.

Eriksen BA, Eriksen CW. 1974. Effects of noise letters upon the identification of a target letter in a nonsearch task. Perception & Psychophysics. 16(1):143-149.

Fox RS, Zhang M, Amagai S, Bassard A, Dworak EM, Han YC, Kassanits J, Miller CH, Nowinski CJ, Giella AK et al. 2022. Uses of the nih toolbox® in clinical samples. A Scoping Review. 12(4):307-319.

Fromm D, Holland AL, Nebes RD, Oakley MA. 1991. A longitudinal study of word-reading ability in alzheimer's disease: Evidence from the national adult reading test. Cortex. 27(3):367-376.

Geffen G, Butterworth P, Geffen L. 1994. Test-retest reliability of a new form of the auditory verbal learning test (avlt). Arch Clin Neuropsychol. . p. 303–316.

Glisky EL, Alexander GE, Hou M, Kawa K, Woolverton CB, Zigman EK, Nguyen LA, Haws K, Figueredo AJ, Ryan L. 2020. Differences between young and older adults in unity and diversity of executive functions. Neuropsychology, development, and cognition Section B, Aging, neuropsychology and cognition. 28(6):895–915.

Malek-Ahmadi M, Nikkhahmanesh N. 2024. Frontiers | meta-analysis of montreal cognitive assessment diagnostic accuracy in amnestic mild cognitive impairment. Frontiers in Psychology. 15.

Moradi E, Hallikainen I, Hänninen T, Tohka J. 2017. Rey's auditory verbal learning test scores can be predicted from whole brain mri in alzheimer's disease. NeuroImage: Clinical. 13:415–427.

Nasreddine Z, Phillips N, Bédirian V, Charbonneau S, Whitehead V, Collin I, Cummings J, Chertkow H. 2005. The montreal cognitive assessment, moca: A brief screening tool for mild cognitive impairment - pubmed. Journal of the American Geriatrics Society. 53(4).

Rogers. 1995. Costs of a predictible switch between simple cognitive tasks. Journal of Experimental Psychology: General. 124(2):207–231.

Rossetti HC, Lacritz LH, Cullum CM, Weiner MF. 2011. Normative data for the montreal cognitive assessment (moca) in a population-based sample. Neurology. 77(13):1272-1275.

Ryan L, Cardoza JA, Barense MD, Kawa KH, Wallentin-Flores J, Arnold WT, Alexander GE. 2012. Age-related impairment in a complex object discrimination task that engages perirhinal cortex. Hippocampus. 22(10):1978-1989.

Stark SM, Kirwan CB, Stark CE. 2019. Mnemonic similarity task: A tool for assessing hippocampal integrity. Trends in cognitive sciences. 23(11).

Stark SM, Yassa MA, Lacy JW, Stark CE. 2013. A task to assess behavioral pattern separation (bps) in humans: Data from healthy aging and mild cognitive impairment. Neuropsychologia. 51(12).

Talboom JS, Håberg A, Both MDD, Naymik MA, Schrauwen I, Lewis CR, Bertinelli SF, Hammersland C, Fritz MA, Myers AJ et al. 2019. Family history of alzheimer’s disease alters cognition and is modified by medical and genetic factors. eLife. 8:e44000.


Information Classification: General
